# Implementation of a Virtual Hospital in the Home Service for Patients With COVID-19 in Queensland, Australia: Mixed Methods Evaluation Using the RE-AIM Framework

**DOI:** 10.2196/73749

**Published:** 2025-09-19

**Authors:** Linh Khanh Vo, Hannah E Carter, Steven M McPhail, Kelly McGowan, Shannon Wallis, Kate Atkinson, Michelle J Allen

**Affiliations:** 1 Australian Centre for Health Services Innovation Faculty of Health, School of Public Health and Social Work Queensland University of Technology Kelvin Grove Australia; 2 Digital Health and Informatics Directorate Metro South Health Woolloongabba Australia; 3 Preventative and Prison Health Services West Moreton Health Ipswich Australia; 4 Hospital in the Home | Multidisciplinary Avoidance Post Acute Service | Acute Medicine and Continuum Care Division of Medicine West Moreton Health Ipswich Australia

**Keywords:** virtual ward, hospital in the home, home-based care, patient remote monitoring, reach, effectiveness, adoption, implementation, and maintenance, implementation science, health economics, digital health, COVID-19

## Abstract

**Background:**

Hospital in the home (HITH) provides home-based care as an alternative to traditional hospitalization. In response to the COVID-19 Omicron wave, a public hospital in the rural Western portion of Southeast Queensland implemented a virtual HITH service to support adults, maternity patients, and children with moderate COVID-19 symptoms and additional health concerns. Although the pandemic accelerated the uptake of virtual care within HITH models, existing literature has focused on clinical outcomes, with limited evidence on key implementation outcomes.

**Objective:**

Using the RE-AIM (reach, effectiveness, adoption, implementation, and maintenance) framework, this study evaluated the implementation of the virtual COVID-19 HITH service and identified factors influencing its implementation, to inform ongoing service development and support potential scaling of this model of care.

**Methods:**

The RE-AIM implementation science framework was selected to guide the evaluation, capturing both clinical and contextual dimensions of implementation at both individual and organizational levels. Quantitative data on service usage and costs were retrospectively extracted from electronic medical records and finance records, while patient experience data were drawn from patient-reported experience measures surveys. Qualitative data were collected through one-on-one interviews with patients and staff. All data sources were analyzed separately and then triangulated within the RE-AIM framework to understand what occurred, how, and why.

**Results:**

The service admitted 3192 patients, most of whom were female (2027/3192, 63.5%), English-speaking (3140/3192, 98.4%), and residing in socioeconomically disadvantaged areas (1879/3192, 58.9%) (reach). The model was feasible and safe to implement, managing 3240 admissions with no reported deaths. Patients valued continuous access to care and described better recovery experiences at home (effectiveness). Staff viewed the model as appropriate for identifying and managing high-risk patients in the community, easing pressure on hospital beds (adoption). The service cost Aus $ 5.4 million (US $3.5 million) over 11 months. Implementation barriers included the urgency of the pandemic scenario, limited infrastructure and human resources, and changing requirements in relation to COVID-19. These were mitigated by several people factors that were critical to its successful implementation, including a consultant-led structure, staff commitment, and adaptability (implementation)*.* The service saved 16,651 inpatient bed days before being integrated into core HITH operations. The experience strengthened staff capabilities in emergency response, virtual care delivery, and strategic planning. The model shows promise for broader application into pediatric care, though further work is needed to enhance interdepartmental collaboration and staff recognition (maintenance)*.*

**Conclusions:**

This study demonstrated that a virtual HITH model can be implemented effectively and safely at scale. Findings support its potential for integration into routine care, provided that adequate resource planning, a skilled and multidisciplinary workforce, well-defined care pathways, and equity-focused strategies are in place.

## Introduction

The growing demand for resource-intensive inpatient hospitalization challenges hospital sustainability [1,2]. Hospital in the home (HITH) presents an alternative by delivering home-based care to patients who require clinical governance, monitoring, or input that would otherwise need treatment in a traditional inpatient hospital bed [3]. Global evidence demonstrated that HITH can achieve equal or better patient outcomes [3-5], improved quality of life [6,7], and substantial cost savings [8-10] compared with conventional inpatient care for selected health conditions.

While the concept of shifting certain aspects of care from the hospital to the home has existed for decades, its implementation accelerated significantly following the onset of the COVID-19 pandemic, primarily due to hospital capacity constraints and new reimbursement opportunities [11,12]. The traditional HITH model of clinicians visiting patients’ homes evolved during the pandemic to incorporate virtual care modalities such as audiovisual teleconferencing and remote patient monitoring [13-15]. These modalities replicate the systems, staffing, and routines of a traditional hospital ward, allowing for the delivery of acute care to patients with COVID-19 who are deemed lower risk but still requiring monitoring for potential deterioration [3], while facilitating physical distancing to minimize exposure risk [16,17].

The literature has documented the implementation of these virtual HITH services [18-20], alongside similar technology-enabled models of remote patient care [21-26] introduced in response to the COVID-19 pandemic across different waves in Australia. While the evidence consistently highlights those models as safe and effective to manage patients with COVID-19 [18,20-22,24,26], there remains limited evidence on other implementation outcomes, which are essential for supporting their broader uptake [27]. Despite an increasing evidence base, widespread adoption and scaling of HITH services have been limited in practice. Reported implementation barriers include the need for more compelling evidence of safety [13], the lack of perceived need [28], digital health equity [18,29], and misaligned or a lack of financial incentives for implementing such a complex intervention that may disrupt traditional clinical practices [19,30,31]. In the Australian context, very few studies have explored these challenges in depth, with existing work limited to pediatric virtual HITH services [18] or services in metropolitan settings [19].

This study presents a pragmatic, mixed methods evaluation of a virtual care–enabled HITH service that delivered hospital-level care to adults, maternity patients, and children with moderate COVID-19 symptoms in Queensland, Australia, during the Omicron wave. Using the RE-AIM (reach, effectiveness, adoption, implementation, and maintenance) framework, we aimed to evaluate key implementation and service outcomes and identify factors influencing its implementation. Findings from this study are intended to inform ongoing service development and guide potential scaling of this model of care to meet rising health care demand and increase inpatient care capacity.

## Methods

### Setting

This study was conducted at a large 350-bed acute care public hospital serving the rural western region of Southeast Queensland, Australia. The region faces unsustainably high demand for hospital services due to having the fastest-growing community in the state, which is projected to nearly double by 2046. It is also culturally and economically diverse, with 30% of residents living in a regional setting and 51% classified among the most disadvantaged [32].

### The Virtual COVID-19 Hospital in the Home Service

In response to the surge in COVID-19 Omicron (sublineage B.1.1.529/BA.1) cases [33], a virtual COVID-19 HITH service was implemented from December 19, 2021, to November 28, 2022. This new service stream was dedicated to supporting patients with COVID-19 with moderate symptoms and additional health concerns, such as pregnancy or chronic conditions. It was managed by the hospital’s existing HITH unit, which routinely delivers hospital-level care at patients’ residences or in the HITH clinic for conditions including skin infections, pneumonia, severe chest infections, urinary tract infections, and the administration of intravenous antibiotics at home.

The new virtual COVID-19 HITH service operated as a consultant-led care model, with patients admitted under a medical consultant. It adopted a “whole family” approach, incorporating pediatricians and midwives to enable coordinated care for parents and children through a single point of contact. Service referrals came from various internal and external sources, with patients able to self-refer via a web-based eligibility checker, hotline, or digitally assisted telephone triage system. Admission criteria were based on the pandemic addendum B to the state-level HITH guideline [34]. In Queensland, HITH provides a comparable level of care to what is provided in the inpatient in-hospital admitted care, which means without this HITH service, the patient would have been admitted to a bed within the hospital setting for treatment as an inpatient. HITH patients are considered hospital inpatients and are funded through the same activity-based funding mechanism applied to public hospital admissions [35]. Admitted HITH patients had to be medically stable, safely self-isolate at home, have an appropriate residence for home-based care (eg, not cohabitating with individuals at high risk of complications), and have access to a telephone.

Referred patients were assessed against these admission criteria and triaged as mild, moderate, or high risk. Admitted patients were contacted via phone or videoconference by a team of doctors and nurses to discuss their symptoms and care plan. Devices such as oximeters and thermometers were provided to patients as needed, with sphygmomanometers only provided to the maternity cohort. Patients were asked to upload their observations into a web-based coordinator system, which was reviewed daily by a nurse for any signs of deterioration. Patients whose symptoms worsened, or who required administration of antivirals and pathology, were transferred to a hospital-located HITH treatment room for in-person review.

### Study Design

This paper is part of a broader research project evaluating this virtual COVID-19 HITH service. A separate publication [36] reported on its effectiveness and cost-effectiveness, while this paper examined implementation outcomes at both individual and organizational levels.

The RE-AIM implementation science framework [37,38] was used to guide and structure the analysis and reporting of this paper. RE-AIM extends beyond traditional measures of effectiveness of an intervention to consider the broader contextual factors that shape how an intervention is implemented in real-world settings [39]. Hence, this framework was selected to facilitate the identification of which components of the virtual COVID-19 HITH service worked well, and which areas may require adaptation to strengthen future implementation and long-term sustainability.

### Data Collection

#### Overview

Four sources of quantitative and qualitative data were simultaneously collected via patients’ electronic medical records (EMRs), internal finance documentation, patient-reported experience measures surveys (PREMS), and patients and staff interviews (Table 1).

**Table 1:** Summary of data sources, types, and timing relative to service implementation.

Data source	Type of data	Time Period	Relation to service timeline
Patients’ electronic medical records	Patient demographics and all emergency department, inpatient, and outpatient encounters within one year following each patient’s index HITH^a^ admission	Dec 2021 to Nov 2023	Full service operational period with 1 year follow-up
Patient-reported experience measures surveys	Patients’ self-reported experiences of how care was delivered during their HITH admission, covering helpfulness, cultural needs, trust in clinicians, involvement in decisions, clarity, respect, contact, and overall care rating	Jan 2022 to Oct 2022	Majority of service operational period (excluding the first and final months)
Internal finance documentation	Direct costs related to clinical and domestic supplies, computer-related services and equipment, pathology, and clinician time	Dec 2021 to Nov 2022	Full service operational period
Staff interviews	Staff perceptions of clinical appropriateness, perspectives on implementation barriers and facilitators, and improvement suggestions	Apr to July 2024	2 years post establishment
Patient interviews	Patient-reported experiences, satisfaction, improvement suggestions, and care recommendations	Jan to July 2024	2 years post establishment

^a^HITH: hospital in the home.

#### Quantitative

Service usage data were retrospectively extracted from routinely collected EMRs by a designated data custodian within the health service, upon request from the research team. Key variables included patient demographics, admission and discharge datetime, intensive care unit admissions, and diagnosis-related group for all patients admitted to the virtual COVID-19 HITH service. The dataset also captured all emergency department, inpatient, and outpatient encounters within 1 year following each patient’s index HITH admission. A complete list of data items requested is provided in Table S1 in Multimedia Appendix 1.

Cost data were extracted from internal finance documentation by research team members who also held staff positions within the health service (KM, SW, and KA). These included costs related to clinical and domestic supplies, computer-related services and equipment, pathology, and clinician time. The same team members also obtained patient experience data from inpatient PREMS collected by the health service from January 2022 to October 2022 (Multimedia Appendix 2). The PREMS was a standardized, statewide web-based survey sent to patients’ mobile phone numbers 2 days after hospital discharge.

#### Qualitative

Qualitative data were collected through one-on-one interviews with both service providers and consumers by a single interviewer (LKV) to ensure consistency. All 49 staff members involved in the service’s development, delivery, and management were invited via email. A total of 200 English-speaking former patients were also invited and mailed a study pack containing participation information. The target sample of 200 patients was selected through stratified random sampling. Patients were randomly selected within each unique stratum defined by age, sex, indigenous status, marital status, and residential suburb, with selection weights applied to reflect the underlying population distribution. Patients flagged as deceased were excluded prior to sampling. The sample size of 200 was determined based on feasibility, given the research team’s available time and budget to prepare and mail the study packs.

Each participant was interviewed once. Staff interviews were conducted via Microsoft Teams, and patient interviews were conducted by phone, in line with their preferred communication methods. The interviews followed a semistructured format guided by the study objectives (Multimedia Appendix 3). Staff were asked about contextual factors influencing service delivery, perceptions of patient safety, and areas needing improvement. Patient interviews sought to cover their overall experiences with the service, including its usability, positive and negative aspects, areas for improvement, and whether they would recommend it to others in similar situations. All participants were free to comment in other areas. The selection of follow-up questions, question order, and phrasing varied according to each participant’s narrative to enable the emergence of participant-led insights, reflecting their varied histories, modes of expression, and foci of experience.

Participants had no prior knowledge of, or relationship with, the interviewer or those undertaking the analysis. Participants were encouraged to speak freely until they indicated they had nothing further to add. Demographic information was collected at the end of each session with participants’ consent. Interviews were audio-recorded, supplemented with handwritten field notes, deidentified, and transcribed verbatim by LVK. Transcripts were then quality checked against the recordings by a second researcher (MJA).

### Ethical Considerations

This study received ethics approval from the West Moreton Human Research Ethics Committee (HREC/2023/QWMS/91529). Governance approval was also obtained from two organizations: (1) a Public Health Act by Queensland Health (grant 91529) authorizing access to quantitative data from the local data repositories, and (2) a site-specific approval by West Moreton Research Governance Office (SSA/2023/QWMS/91529) permitting the conduct of qualitative interviews at the study site.

A waiver of consent for the use of retrospective quantitative data was approved on the grounds that no active patient participation was involved, all data had been previously collected for routine clinical purposes, and all data were deidentified prior to being provided to the research team.

For the qualitative component, all interview participants were provided with a plain-language information sheet and were asked to return their written informed consent prior to participation. Patient participants received an Aus $ 20 (US $13.196) voucher in recognition of their time and contribution. No compensation was provided to staff participants. The site-specific approval formally recognizes the health service’s provision of in-kind support, including staff time to assist with data collection and participation in this study.

All data were deidentified. Data were shared via a secure, password-protected file transfer platform (Kite works) and stored on encrypted, password-protected hard drives accessible only to members of the research team.

### Analyses

#### Quantitative

Descriptive statistics were used to summarize all quantitative data in RStudio (version 4.3.1; Posit Software, PBC) [40]. Categorical variables are reported as frequencies and percentages, while continuous variables are presented as median with IQR. Service usage data were used to estimate key clinical outcomes, including length of stay, ward transfer rate, discharge rate, 28-day readmission rate, and in-hospital mortality rate. Total hospital bed-days saved were calculated by summing the length of stay across all patients admitted during the service operation period, used to calculate total hospital bed-days saved for the total number of patients admitted during the service operational period. Cost data were used to estimate the average cost per bed day and per patient separation. All costs were inflated and reported in 2024 Australian dollars using an index of hospital price inflation [41]. Patients’ experience data from PREMS, comprising 8 Likert-scale questions (ranging from “most positive” to “most negative”), were summarized by calculating the percentage of responses within each category for each question.

#### Qualitative

Qualitative data were analyzed using an iterative, inductive thematic approach [42], beginning with data familiarization and researcher reflexivity. The semistructured interview guide was adapted over time as new insights emerged. Initial codes were generated and repeatedly revised and reorganized before being collated into potential themes. These candidate themes were then refined and mapped to the 5 RE-AIM dimensions using framework analysis. An essentialist approach was applied in the thematic analysis, reporting experiences, meanings, and reality as perceived by participants. All interview transcripts, including patient letters, were coded by 2 members of the research team (LKV and MJA) using NVivo (version 14; QSR International Pty Ltd) [43]. LKV is a PhD candidate in health services research with mixed method training, and MJA is an experienced qualitative researcher specializing in implementation science. Both researchers independently coded all interview transcripts, after which the coding was compared for consistency. Any discrepancies were discussed and resolved through consensus, and the coherence of emerging themes was further refined through discussions with the broader study team.

Each data source was analyzed separately and triangulated within the RE-AIM framework (Table 2) to offset challenges with data availability and reliability [44], and to provide a more comprehensive picture of not only what occurred, but also why and how both intended and unintended outcomes transpired [45,46].

**Table 2:** Reach, effectiveness, adoption, implementation, and maintenance (RE-AIM) evaluation dimensions of the virtual COVID-19 hospital in the home service.

RE-AIM dimension	Key question guiding the analysis	Operationalization	Data sources
Reach individual	Who were the patients admitted to the service?	Number and type of patients admitted Patients’ demographics Awareness and barriers to access	EMRs^a^ Patient interviews Staff interviews
Effectiveness individual	How feasible, safe, and acceptable was the service for patients?	Feasibility (whether patients and health providers could interact through the model of care, eg, number of daily admission volume, peak load, length of stay, staff actions to deliver remote care) Safety (whether the service maintained patient safety, minimized risk, and achieved appropriate clinical outcomes, eg, mortality rate, transfer rate, readmission rate, patient-reported perceptions of safety) Acceptability (whether patients found the service satisfactory and aligned with their expectations, eg, patient-reported satisfaction, perceived benefits)	EMRs PREMS^b^ Patient interviews Staff interviews
Adoption organizational	To what extent was the model taken up by staff and perceived as appropriate?	Extent of staff uptake Appropriateness (whether staff perceived the service as clinically appropriate and valuable at a system-wide level) Facilitators and barriers to adoption	Staff interviews
Implementation organizational	How was the service delivered in practice, and to what extent was it delivered as intended?	Initial service setup and context Fidelity (whether service was delivered as intended) Facilitators and barriers to implementation Cost associated with the establishment and implementation	Patient interviews Staff interviews Finance documentation
Maintenance organizational	To what extent has the service become part of routine practice?	Degree of service institutionalization Impact (whether the service contributed to hospital efficiency, eg, bed-days saved, workforce development) Areas for service extension and improvement	Staff interviews

^a^ERM: electronic medical record.

^b^PREMS: patient-reported experiences measures surveys.

## Results

### Overview

Seven staff members and 9 patients were interviewed between February and August 2024. Two invited patients returned signed informed consent forms but did not participate in interviews. Instead, they submitted letters expressing their views, which were treated as transcripts and analyzed using the same approach for all qualitative data. The response rate was 14% for staff and 6% for patients.

All interviewed staff members were female, including 1 medical consultant, 1 divisional leader, 1 nurse unit manager, 1 clinical nurse consultant, and 3 registered nurses, with specialties in geriatrics, oncology, and pediatrics. Patient participants were predominantly of English or Australian descent, with one identifying as Fijian-Indian. Six of the 9 patients were female, and 5 patients were older than 60 years, with the exception of a younger woman from the maternity cohort who shared experiences related to both herself and her admitted children.

### Reach

During the 11-month operational period, a total of 3192 patients were admitted to the virtual COVID-19 HITH ward, including 2417 adult patients (75.7%), 495 pediatric patients (15.5%), and 280 maternity patients (8.8%). The demographic characteristics of the patient cohort are described in Table 3. The majority of patients admitted were female (2027/3192, 63.5%) and English-speaking only (3140/3192, 98.4%). The relatively high levels of sociodemographic disadvantage observed in the patient cohort reflect the service’s reach into disadvantaged communities, consistent with the socioeconomic profile of the broader geographical catchment (Figure S1 in Multimedia Appendix 1).

**Table 3:** Demographic characteristics of the virtual COVID-19 hospital in the home patients (N=3192).

Baseline characteristics		Values, n (%)
Female		2027 (63.5)
**Age (years)**		
	<5	252 (7.9)
	5-19	295 (9.3)
	20-24	122 (3.8)
	25-39	771 (24.2)
	40-64	1094 (34.3)
	65+	651 (20.4)
	Unknown	7 (0.1)
Aboriginal and Torres Strait Islander		233 (7.3)
Speaking English only		3140 (98.4)
**Marital status**		
	Never married	1069 (33.5)
	De facto/ married	1612 (50.5)
	Separated/ widowed	321 (10.1)
	Unknown	190 (5.9)
Having private health fund cover		1416 (44.4)
**SEIFA^a^- IRSAD^b^ quintile**		
	1 (most disadvantaged)	1879 (58.9)
	2	107 (3.4)
	3	675 (21.2)
	4	504 (15.8)
	5 (most advantaged)	11 (0.3)
	Unknown	16 (0.4)

^a^SEIFA: Socioeconomic Indexes for Areas.

^b^IRSAD: Index of Relative Socioeconomic Advantage and Disadvantage.

Factors influencing Reach included fear and uncertainty; availability of information about the service; the referral process; health literacy; and mobile phone access.

Referrals to the service followed the trajectory of COVID-19 cases, increased significantly after Queensland’s border opening, and declined as case numbers subsided. Interviewed patients emphasized the need for professional health support, driven by fear and uncertainties during the pandemic. Despite substantial demand, most patients were generally unaware of the existence of this virtual COVID-19 HITH service or what it entailed, even after being admitted. They highlighted the need for more transparent community information on accessing the service.

I wasn't aware that it actually existed, basically. And it wasn't explained to me what it was. So I didn't actually know why people were ringing me to start with […] I wasn't even aware that they were from the hospital.

Patient 8

Frequent changes in the referral process created barriers to service access. Staff expressed concerns about delays with the self-referral platforms, as patients’ information often arrived 3-4 days after their positive tests, by which time they may have begun recovering. A few staff emphasized that the eligibility criteria listed through those platforms occasionally led to oversights, with some patients requiring hospital-care level care registered but not referred, and others with low-acuity needs unnecessarily admitted.

Sometimes I would call an at-risk family that was classed through the website and they would ask me to call their family, their cousin, their neighbour, because they also had COVID.

Staff 3

Concerns were also raised about patients with limited health literacy or cognitive impairments who may not recognize when they need care. Technological barriers further complicated access for patients without mobile phones or those unable to answer calls at unscheduled times.

### Effectiveness

The virtual COVID-19 HITH service was feasible to implement and operate at scale. Over the course of the service, 3240 admissions were recorded among 3192 patients, with 44 patients admitted more than once. The daily number of admissions fluctuated from 2 in early January 2022 to 43 in mid-July 2022. During peak periods (mid-March and mid-July 2022), the team successfully managed up to 120 patients simultaneously. The median length of stay in the virtual COVID-19 HITH ward was 5 (IQR 4-6) days.

The model of care was also found to be safe, with no adverse events observed in both clinical records and patient reports. Twelve patients (0.4%) required escalation to a physical hospital bed, none of whom required intensive care, and no deaths occurred either in the virtual HITH ward or following escalation. Detailed clinical outcomes are reported elsewhere [36].

Interviews further confirmed that the virtual COVID-19 HITH service provided a safe and effective alternative to hospital-level care for managing COVID-19 symptoms at home. All interviewed patients, with a length of stay ranging from 2 to 21 days, reported positive experiences and no clinical deterioration. All of them had comorbidities or health concerns requiring regular medications and monitoring, including chronic conditions, recent surgery, breathing issues, scarring from previous pulmonary embolisms, and pregnancy complicated by caring for young children. Staff reported routinely checking that patients’ environments were safe for self-isolation, ensuring they had access to essential support and could maintain overall well-being. If patients could not be reached by phone, text messages were sent as a follow-up. In extreme cases, as one staff member explained:

Sometimes you couldn't get on to people. They wouldn't answer their phones, and, you know, I think a few times we did have to do welfare checks on people who lived alone. If you just couldn't get on to them all day and then the next day, then we would have to call the police and ask them to do a welfare check.

Staff 4

**Figure 1:**
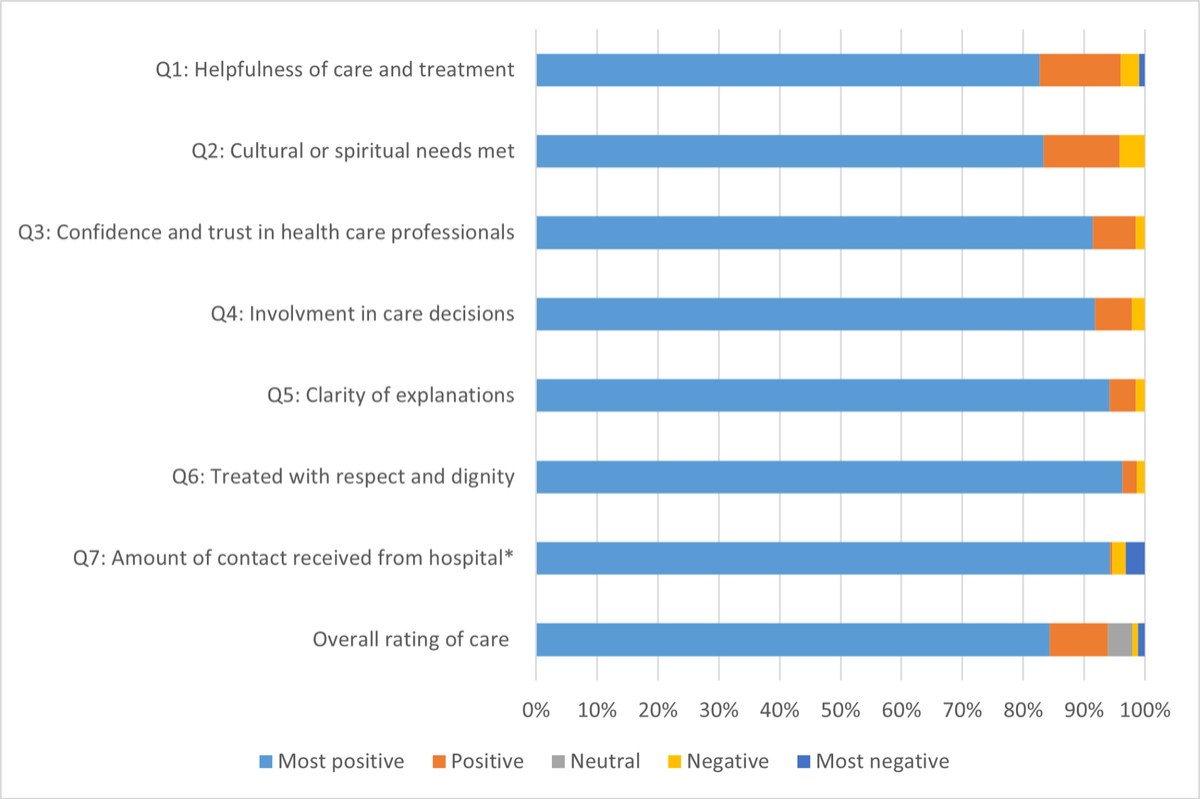
Patients’ reported experiences of using the virtual COVID-19 hospital in the home service (n=599). Data were extracted from a state-wide web-based survey, inpatient patient reported experience measures survey (PREMS) from January 2022 to October 2022. *Q7 had a 3-point scale, which has been converted: most positive (the right amount), negative, too much, and most negative (not enough).

Figure 1 presents PREMS results from patients who used the virtual COVID-19 HITH service. Predominantly, responses were “most positive” across all categories, indicating a high level of acceptability and patient satisfaction in 7 domains of quality of care.

Patient interviews confirmed PREMS’ results that the virtual COVID-19 HITH service was user-friendly and highly satisfactory. Factors influencing acceptability included the quality of staff interactions and the social and emotional benefits of the home environment. Patients particularly valued the quick response times and the respectful, caring attitude of the clinical staff during their admission.

Whenever I rang, someone always answered. I didn't get an answer machine. And I didn't have to wait for someone to ring me back. Somebody answered the phone every time.

Patient 9

I know what doctors and nurses are like, but they were actually very caring. They asked if I wanted more phone calls towards the end when I was feeling quite well. They always asked if I was happy with what I had, or would I like them to do more couple of days?

Patient 8

Several patients noted that receiving care at home contributed to a better recovery experience compared with staying in a physical hospital bed. They emphasized the benefits of remaining close to loved ones and the positive impact on emotional and mental well-being when receiving care in a familiar environment. One patient, who had experienced COVID-19 treatment both in a physical hospital ward and through the virtual HITH service, reflected:

I actually had COVID twice and got into the hospital with it as well… Home thing is a lot better. You know, I felt a lot more restful, and I felt that I recovered quicker at home because I don't have the continual noise that goes on at a hospital

Patient 5

All participants expressed that they would strongly recommend the service to others in similar situations. Many also voiced a strong desire for more of this type of service in the community and a willingness to receive more health care at home, provided their symptoms remained stable.

### Adoption

Given that the service was implemented out of necessity in rapid response to an escalating pandemic scenario, all staff in the existing HITH unit adopted the model, with many transitioning from other hospital wards to meet rising demand. Despite limited prior experiences with virtual care, staff were broadly supportive of the virtual HITH approach for managing patients with moderate-severity COVID-19.

Attitudes and composition of staff were seen as enabling factors. All staff demonstrated a strong commitment to delivering high-quality care, facilitated by strong advocacy from clinical leaders and cohesive teamwork. Staff perceived the new virtual HITH model as clinically appropriate in identifying and managing patients at high risk of deterioration in the community, while reducing hospital bed occupancy and alleviating system-wide pressures. They highlighted the importance of having on-site doctors to support rapid clinical decision-making and timely prescription of medications, further safeguarding patient health. As a consultant-led care model, it achieved quicker patient turnover compared with nursing-led services in nearby hospitals.

From a system wide perspective, I've seen the significant pressure on beds and staffing. And we know that patients will present to a hospital if they're concerned […] And I think we really did achieve what we set out to achieve in preventing multiple anxious patients with COVID rocking up to the hospital because they're very concerned. Since I've been here, it opens my eyes to much more of the potential in this space to help relieve the bed pressures in the system.

Staff 7

However, frontline staff reported feeling overwhelmed and exhausted. This was expressed in relation to the broader strain on health care workers during the pandemic and the pressure of adapting rapidly to a new model of care. Key implementation barriers, including limited preparedness, fluctuating patient volumes, and rapidly evolving clinical guidelines, contributed to the difficulties of maintaining a balanced workload.

There was a lot of emotion, I would say. I've never seen the HITH nurses feeling that overwhelmed. And really, I think not one of us didn't cry going home because it was so hard. It was something none of us want to go through again because there were so many facets to it.

Staff 5

### Implementation

#### Overview

The implementation of the virtual COVID-19 HITH service was challenged by the pace required to set up the service, limitations related to the availability of both infrastructure and human resources, and changing requirements in relation to COVID-19 and associated eligibility criteria. However, this was somewhat counteracted by a strong commitment by staff to deliver a quality service, by working flexibly and adapting the service to meet changing requirements.

#### Early Establishment

The announcement of Queensland’s border reopening, following an extended period of interstate restriction, was the key trigger prompting immediate actions to establish a dedicated virtual COVID-19 HITH stream. Service design was informed by internal nurse-led virtual care models for managing patients with COVID-19 in earlier waves and those with chronic conditions, and external responses to cruise ship outbreaks and early cases at another tertiary hospital. Protocols for antiviral administration were informed by interviews with clinicians from another state where such treatments were already in use. Additional details on the initial planning phase of the service are provided in Multimedia Appendix 4.

The launch of the new virtual COVID-19 HITH stream led to a temporary suspension of other standard HITH services to allow the entire HITH team to manage the massive influx of patients. While some early groundwork was in place, staff reported that the service was launched rapidly without sufficient preparation and capacity in the early stage, placing significant strain on operations. Redeployed staff from other wards with no or little prior experience with virtual care required rapid on-the-job training, supported informally by the core HITH team.

It’s very quickly became that HITH was running virtual COVID and nothing else. In an ideal world, if we had had a team prepped, ready to go, that would have been really beneficial. Instead, we found ourselves rapidly pulling from outpatient departments that had been temporarily closed to start staffing the model as quickly as we could to reduce the impact on other HITH services, because that was also important to keep running at the time.

Staff 7

Recruitment of additional staff began approximately one month after the service rollout. The onboarding of new staff provided much-needed relief and enabled the resumption of other HITH services alongside the new virtual COVID-19 HITH stream. Pediatric and maternity staff were not involved in the initial phase but joined the team later.

The service also faced infrastructure challenges. It operated without a purpose-built workspace and sufficient equipment, further complicated by physical distancing and personal protective equipment requirements. The team was initially assigned a temporary cabin outside the hospital, then moved into a small clinic space within the hospital. Some frontline staff expressed frustration over delays in securing essential resources needed to deliver the service and the late inclusion of key personnel in the early planning stages.

We were in an old dialysis urology, just a little section downstair on 5G that we were using as a little clinic space. A lot of areas in the hospital had closed down with people working from home, and we had to go to different levels and spread out. There wasn't a real dedicated place for us. We had to find our own space to make all the phone calls and then were kicked out and then had to find another place […] It was so hot … our nurses had to gown up in COVID clothes […]. You had to sit there for hours, […]. You had to leave and doff off to get a drink.

Staff 5

It was acknowledged that creating a business case for committing resources and allocating staff was particularly challenging due to uncertainty around the requirements of a new model of care, and competing organizational priorities during an already difficult time. As one participant who was in a leadership role said:

It's very hard for a health service to provide budget and staffing around a model that is unknown. So as much as it may have seemed like we weren’t listening, there’s also a budgetary concern about committing FTE and resources to something unknown. We didn't know what we were going to need. On reflection of that and on probably lessons learned, it's looking at the base resources that you need to prepare the model efficiently, with a dedicated plan in place for what resources you will use when the model needs to be implemented.

Staff 6

#### Fidelity

Staff demonstrated remarkable flexibility in responding to implementation barriers and addressing diverse patient and operational needs. One key area of adaptation was the referral pathways and admission process. In the first 2 weeks, all referrals were accepted from local general practitioners and internal sources, including the public health unit, other hospital wards, emergency departments, and outpatient clinics. As demand increased, self-referral mechanisms were introduced. The initial service design was modeled after existing chronic disease management programs, anticipating a large volume of patients requiring active home monitoring. Although admission criteria focused solely on moderate cases requiring clinical support, limited oversight, particularly from self-referral platforms, led to a high volume of referrals from individuals with low-acuity symptoms, and admitting all of them creates a substantial administrative burden:

Admitting all of them like we did from the beginning, every single patient notified were admitted, we then had to go back and cancel all of these admissions after we assessed them because we realised, they didn't need us, or they didn't need that level.

Staff 6

The service subsequently shifted to reviewing all referrals prior to admission. Despite this adjustment, the high volume of incoming referrals continued to strain administrative resources. Additional complexity emerged with the inclusion of pediatric and maternity patients due to their distinctly different clinical parameters and risk profiles. The use of the web checker, which generated the bulk of referrals, was eventually decommissioned in late July 2022.

The amount of administrative resources required to run a service like this were significantly underestimated. There’s a big difference between aiming to avoid hospital presentations for low-acuity COVID-positive patients, and substituting hospital care for those who are acutely unwell with COVID. This distinction is where the administrative workload comes in. We had to quickly create outpatient encounters for all screened patients and then admit only those who required higher-level care.

Staff 7

Remote patient monitoring protocols were also adapted to reflect both patient capabilities and logistical realities of setting up the web-based coordinator platform. While most patients received the remote monitoring devices, only those who were technologically proficient uploaded their measurements directly to the web-based platform, while others could opt to report them through phone consultations. Additionally, when there were delays in configuring access to the web-based platform, staff collected patients’ measurements by phone to avoid postponing care. The service was eventually transitioned from predominantly equipment-based remote patient monitoring to self-reported symptoms over the phone.

We provided remote patient monitoring equipment to many patients, but often gathered results over the phone because the online platform couldn’t handle the quick turnover of numbers we were managing. If we knew we had someone for longer, or we were very concerned and needed to monitor them very closely, we would onboard them to the online platform.

Staff 7

These adaptations affected the service fidelity, in that it was delivered differently than originally intended. However, such flexibility was necessary to accommodate the contextual flux and changing service demands.

#### Cost

The total cost for the 11-month service operating period was Aus $5.4 million (US $3.56292 million), translating to an average of Aus $439 (US $289.6522) per patient bed day and Aus $2255 (US $1487.849) per separation. Labor costs accounted for 93% (Aus $5 million/5.4 million [US $3.299 million/3.56292 million]) of total expenditure. Further details on the costing methodology and a modeled cost-effectiveness analysis comparing the virtual COVID-19 HITH service with conventional inpatient care are reported elsewhere [36].

### Maintenance

Following a substantial decline in COVID-19 referrals, the virtual COVID-19 HITH service was decommissioned and integrated into the standard workflows of the HITH team on 28 November, 2022. Patients with COVID-19 meeting clinical criteria continue to be managed by the team alongside patients with other conditions. The choice of virtual, face-to-face, or a hybrid care is now applied universally across all HITH patients, regardless of diagnosis. Virtual platforms are used to monitor clinically stable patients at home more efficiently, while in-person visits remain important for tasks requiring physical presence, such as assessing dietary habits, home setup, or other factors that may impact patient outcomes.

Beyond immediate clinical benefits, the service saved a total of 16,651 inpatient bed days from its launch to decommissioning. The service also strengthened organizational capacity to respond to future health care shocks. Staff described it as a steep but valuable learning curve that enhanced their capacities in service management, strategic planning, quality improvement, and leadership, building confidence in managing similar challenges in the future.

The implementation of this service also had a lasting impact on workforce skills in delivering virtual care models.

I'm currently now starting a tele-chemo service. So I guess that the experience working in the COVID here, virtual, has really helped in the setup of this new service. I think the time that was spent there was very valuable for me to give another perspective on how treatment can be delivered to patients without them being in here, paying up space in the hospital for other more acute patients.

Staff 4

As the HITH unit continues to grow beyond its original catchment area, with plans underway to expand into pediatric care, staff have identified several key areas for service improvement. First, strengthening interdepartmental collaboration is seen as essential to ensure all HITH patients, though not physically present in the hospital, receive equitable access to care. This includes timely access to medical imaging and specialist consultations, comparable to patients in a physical ward. Second, addressing skepticism towards virtual care among new staff and patients was highlighted, with suggestions to incorporate a brief virtual care introduction into staff onboarding and education sections during patients’ first consultations. Finally, a strong desire for greater organizational recognition and voice was emphasized. This acknowledgment is seen as crucial for securing additional funding to meet growing demand, acquire necessary equipment, and develop skills in virtual care delivery.

I think a lot of the work that HITH does generally is really unseen. And because our patients are at home, it's almost like out of sight, out of mind. Thinking that how much work goes into keeping these patients out of hospital, and the resourcing that's required to maintain and support a model like that, I don't think our team got the right kind of acknowledgement.

Staff 7

## Discussion

### Principal Results

This study demonstrated the successful implementation of a virtual COVID-19 HITH service using the RE-AIM framework. The model of care was shown to be both feasible and safe for delivering hospital-level care at home for patients with moderate COVID-19 symptoms and additional health concerns. Patients highly valued the service as a reliable means of accessing timely and continuous care during a period of uncertainty, while experiencing the comfort and psychological benefits of recovering at home. Staff viewed the model as effective in remotely supporting patients at high risk of deterioration, thereby preserving critical hospital resources. The service also saved a substantial number of inpatient bed-days and served as a learning opportunity that built workforce capability and confidence in responding to future health emergencies and delivering virtual care.

The evaluation highlights several people factors that were critical to its successful implementation, including rapid clinical decision-making through a consultant-led structure, staff commitment, and adaptability in the face of challenging and changing circumstances. The urgency of COVID-19 scenario also served as a catalyst, prompting rapid pivots from traditional home visits, HITH models, and staff upskilling in virtual care delivery. However, the dynamic and unpredictable nature of the pandemic also introduced significant implementation barriers. Constantly changing government requirements and fluctuating patient volumes resulted in inadequate staffing capacity in the early stage, along with frequent changes to clinical governance processes throughout the operational periods. These pressures were further intensified by competing priorities within a resource-limited environment, making it difficult to staff and establish infrastructure for such a large-scale service in a short period of time.

### Comparison With Prior Work

The service’s demonstrated effectiveness and high patient satisfaction are consistent with findings from traditional HITH home-visit models in the literature [47-50], as well as other pandemic response strategies involving home monitoring of patients with COVID-19 during concurrent [18,22,23] and previous waves [21,24-26]. This study builds on that evidence by demonstrating similar outcomes from a combined virtual and bed-replacement model across a more diverse cohort of adults and pediatrics, encompassing whole family units in some cases. Importantly, the implementation of this model of care was also demonstrated to be successful in a regional population characterized by significant sociodemographic disadvantage, extending the currently limited Australian evidence base, which has predominantly focused on pediatric populations [18,20] or in metropolitan settings [19,51].

Factors influencing the implementation, such as leadership support, staff commitment, workforce flexibility, alongside technological limitations, digital health equity issues, and resource and funding constraints, align with previous research [18,19,29-31,52]. Using the RE-AIM framework, this study adds a deeper understanding of the operational complexities involved in navigating service implementation in the context of an emergency response. It also identifies practical strategies to support quality HITH care during challenging times, including active follow-up to ensure patients receive care safely from a distance, real-time modifications to practices in response to evolving government directives and patient needs, flexible communication channels to mitigate digital exclusion, and simplifying the use of technologies to keep all patients connected to care.

### Implications

This study highlights several pandemic-specific factors that created unique conditions requiring short-term adaptations, including the temporary suspension of other HITH services and rapidly evolving governance processes. However, many findings extend beyond the pandemic context. The model’s effectiveness, high patient satisfaction, and substantial bed-day savings suggest strong potential for scalability, not only during future health system shocks but also in routine care, particularly as health systems face rising demand and budget constraints [1]. Its successful implementation in managing patients with diverse health needs further supports its scalability beyond the adult populations, with potential to be extended to pediatric and maternity care.

The eventual decommissioning of this dedicated COVID-19 virtual HITH stream, and its integration into the business-as-usual of the HITH unit, reflects both the time-limited nature of COVID-19–specific demand and the value of virtual care in expanding traditional HITH home-visit models. Our findings support the adoption of a flexible, blended care model that combines virtual and in-person visits tailored to each patient’s clinical and contextual needs. Consistent with previous research [53-55], this approach improves care by capturing more clinical data points than home visits alone and optimizes staff workflow by reducing travel time, while still allowing in-person assessments when needed. Our findings reinforce the importance of recognizing virtual care modalities as an integral component of modern HITH delivery rather than merely an adjunct.

To support the transition of this virtual HITH model from temporary solutions into enduring components of health system delivery, this study emphasized the importance of adequate planning and investment to establish the base resources and maintain a strong multidisciplinary staffing profile capable of meeting diverse patient needs. Future iterations should incorporate a robust data aggregation system and establish clear governance pathways to improve patient selection and reduce administrative burdens. The development of shared decision-making tools, as shown to be effective in a similar context [56], may further enhance these processes. Finally, equity should remain a core principle. Strengthening interdepartmental collaboration is needed to ensure all patients, even when receiving remote care in a HITH setting, have timely access to other health services if referred or required. Whilst our findings demonstrated successful implementation of the service in disadvantaged and regional populations, the predominance of English-speaking patients suggests a need to develop inclusive communication strategies to better engage culturally and linguistically diverse communities.

### Strengths, Limitations, and Future Recommendations

This study leverages the strength of multiple data collection methods to capture quantitative outcomes from a large number of patients, while gaining deeper insights through individual interviews. By using postal recruitment rather than web-based methods, we were able to include voices from older people with low digital literacy and avoid inherent bias. Staff interviews featured perspectives from nearly all frontline roles, as well as staff in leadership positions who were instrumental in the setup of the service. Furthermore, although conducted within a single hospital, this site is the largest tertiary facility in the region, covering a large catchment area spanning urban and rural locations, which enhances the generalizability of our findings to more settings.

The study has some limitations. First, comorbidity information was not recorded in the EMRs database, limiting our ability to assess how different health conditions might impact patients’ access to and safety within the service. Second, as with all patient-reported data, there may be some self-selection and recall bias. Our patient sample was not diverse in language and ethnicity, and recalling experiences from 2 years ago may have posed challenges for some participants. Last, as this service was introduced out of necessity in response to a health crisis, we could not determine how uptake might differ if patients had a choice between virtual and in-person HITH care. While this study showed patients’ and staff’s willingness and ability to engage with the care model, some staff noted initial skepticism among patients, but we were unable to recruit those who held these reservations. We recommend further investigation into factors influencing HITH service rejection in contexts similar to our study, building upon existing US-based research [52]. Future research should also focus on developing a better understanding of patient preferences to inform the development of more inclusive virtual HITH services. Critical areas for investigation include evidence-based decisions regarding which modalities to offer, for which populations, and in what circumstances. Particular attention should also be paid to equity considerations, especially in identifying strategies to better engage culturally and linguistically diverse communities and those who are less digitally literate.

### Conclusions

Recent advancements in remote monitoring technology, coupled with the pressure on health systems caused by the COVID-19 pandemic, have motivated many countries to prioritize HITH services. This study demonstrates the successful implementation of a virtual HITH service in Queensland, Australia, which offered a safe alternative to inpatient care for diverse cohorts of patients with COVID-19 during peak Omicron variant surges. Our findings support the potential for expanding this model of care beyond the pandemic and into other clinical domains, given its substantial positive impact on patient outcomes and hospital efficiency. Careful resource planning, well-defined care pathways, a skilled and multidisciplinary workforce, and inclusive strategies to ensure equity in access to care are essential for facilitating widespread adoption and scaling of the virtual HITH model from an emergency response measure into routine care delivery.

## Appendix

Multimedia Appendix 1 Additional figures and tables.

Multimedia Appendix 2 Patient-reported experience and outcome measures (PREMS) information sheet (for inpatient and hospital care).

Multimedia Appendix 3 Semistructured interview guide.

Multimedia Appendix 4 West Moreton Health's initial Virtual COVID-19 Hospital in the Home Service Planning Report.

## Abbreviations

EMR: electronic medical record
HITH: hospital in the home
PREMS: patient-reported experiences measures survey
RE-AIM: reach, effectiveness, adoption, implementation, and maintenance

## Data Availability

The datasets generated or analyzed during this study are not publicly available due to the sensitivity and confidentiality of participants’ information, but are available from the corresponding author on reasonable request, subject to approval by the West Moreton Hospital and Health Service and compliance with relevant governance guidelines.
