# Supplementary material for: Implementation of a Virtual Hospital in the Home Service for Patients With COVID-19 in Queensland, Australia: Mixed Methods Evaluation Using the RE-AIM Framework
Source: J Med Internet Res. 2025 Sep 19;27:e73749. doi: 10.2196/73749 (PMC12495369; doi:10.2196/73749)
Supplement: Multimedia Appendix 1 [file jmir_v27i1e73749_app1.docx]

Multimedia Appendix 1

Table S1: List of health utilization data items requested from West Moreton Health patients’ electronic medical record databases

| Data items | Variables |
| --- | --- |
| Admitted patient data | Age (5-year grouping)  Sex  Indigenous status (flag: indigenous/non-indigenous)  Marital status  English speaking status (flag: non-English speaking background)  Postcode of residence  Healthcare card (if available)  Date of admission  Date of separation/discharge  Location of Transferred Care/Discharge destination (flag is Escalated care to ED presentation vs discharged from service)  Flag if patient deceased  First outpatient appointment date (date patient screened)  Admission Date  Plan of care (flag service delivery type)  Clinical risk level on admission (very low, low, moderate, high, very high)  Elective patient status  ICU admission (Y/N)  ICU length of stay  Mode of separation  Continuous episode of care flag  Care type  28-day readmission status (flag yes/no)  Australian-Refined Diagnostic-Related Group (AR-DRG)  Major Diagnostic Category  ICD-10 Diagnosis codes  ICD-10 procedure codes  Facility number  Socio-economic Index for Areas  Accessibility and Remoteness Index of Australia (ARIA) |
| Emergency Department data | Facility number  Triage category  Presentation date  Length of episode (HHMM)  Urgency related group (URG)  Mode of arrival  Visit type  Episode end status |
| Non-Admitted patient data | Service event date  Corporate clinic code  Tier 2 clinic code  Service delivery mode  Care type  New/review flag |

Table S2: Illustrative quotes reflecting key factors associated with each RE-AIM dimension

| **RE-AIM Dimension** | **Illustrative Quotes** |
| --- | --- |
| Reach | **Fear and uncertainty**  *“All you heard was the horror stories about how ill some people were, and I was concerned what would happen with me living alone in almost the countryside” (patient 6)*  *“At the time, I was into my second trimester. I was just really sick. I had a toddler and an older child. The older child tested positive and had some mild symptoms. The toddler, we couldn't test obviously because he was too young. My toddler was quite sick as well…” (patient 7)*  **Availability of information about the service**  *“I wasn't aware that it actually existed, basically. And it wasn't explained to me what it was. So I didn't actually know why people were ringing me to start with… Having COVID, you just don't know what's going on, and it was a bit scary. I wasn't even aware that they were from the hospital. So, I was like, why are they ringing?” (patient 8)*  *“It would be good to have more information of availability of service, types of service, how to access this service, eligibility criteria, cost if any.” (patient 2)*  **Changing requirements in relation to COVID-19 (barrier)** *“Sometimes I would call an at-risk family that was classed through the website and they would ask me to call their family, their cousin, their neighbour, because they also had COVID.” (staff 3)*  **Health and digital literacy (barrier)**  *“There are people out there who are not educated to know that their health is deteriorating. We would have to give them all that advice. It wasn't just normal everyday people like you and me, we're talking about people that have cognitive deficits, or people that are elderly.” (staff 5)*  *“For elderly people who don’t have a mobile phone, it was hard for them to get to the phone when we ring, because there wasn’t any set kind of time.” (staff 4)* |
| Effectiveness | **Feasibility**  *“I really appreciated that they actually sent the package, I didn't have an oximeter ... and I really appreciated that there was contact every day as well.” (patient 9)*  **Safety**  *“I would say it is probably even more safe that they're on the service at home, rather than no service because, you know, it's the safety net. If they're having shortness of breath and if they're not on the service, by the time they eventually can't breathe and present to ED and are very sick, you know, you can safety net that and potentially get something prescribed to prevent that happening.” (staff 4)*  *“Sometimes you couldn't get on to people. They wouldn't answer their phones, and, you know, I think a few times we did have to do welfare checks on people who lived alone. If you just couldn't get on to them all day and then the next day, then we would have to call the police and ask them to do a welfare check.” (staff 4)*  **Acceptability/ Patient experiences**  *“Whenever I rang, someone always answered. I didn't get an answer machine. And I didn't have to wait for someone to ring me back. Somebody answered the phone every time.” (patient 9)*  *“I know what doctors and nurses are like, but they were actually very caring. They asked if I wanted more phone calls towards the end when I was feeling quite well. They always asked if I was happy with what I had, or would I like them to do more couple of days?” (patient 8)* |
| Adoption | **Staff attitude (enablers)**  *“My staff worked so hard over that Christmas. My staff were like 110%. We all got together and everybody just did what they needed to do to respond. The teamwork was amazing.” (staff 1)*  *"From a system wide perspective, I've seen the significant pressure on beds and staffing. And we know that patients will present to a hospital if they're concerned […] And I think we really did achieve what we set out to achieve in preventing multiple anxious patients with COVID rocking up to the hospital because they're very concerned. Since I've been here, it opens my eyes to much more of the potential in this space to help relieve the bed pressures in the system." (staff 7)*  **Emotional toll**  *"There was a lot of emotion, I would say. I've never seen the HITH nurses feeling that overwhelmed. And really, I think not one of us didn't cry going home because it was so hard. It was something none of us want to go through again because there were so many facets to it." (staff 5)* |
| Implementation | **Urgency of COVID-19 scenario (enabler/ barrier)**  *"Two days after the borders opened, [staff was informed that] a patient is stepped down to our virtual care ... [staff had to] set up a model within 24 hours." (staff 1)*  *"It was a lot of early preparing. However, when the time actually came, there was a lot of planning happening on the fly as things were unfolding.” (staff 7)*  **Infrastructure and human resource shortage (Barrier)**  *“… we had not enough staff [initially]. We had 300 to 400 patients, and our team had not increased by a single staff member” (staff 1)*  *“It’s very quickly became that HITH was running virtual COVID and nothing else. In an ideal world, if we had had a team prepped, ready to go, that would have been really beneficial. Instead, we found ourselves rapidly pulling from outpatient departments that had been temporarily closed to start staffing the model as quickly as we could to reduce the impact on other HITH services, because that was also important to keep running at the time.” (staff 7)*  *"We had to learn the system very fast, logging into a platform and finding the templates. And then it became just second nature. You just come in, you did your job." (staff 5)*  *"It did require me to learn how to assess patients in a different way… I’ve always been in ward environments, where I could visually assess my patients. So having to assess them without seeing them, that was difficult at the start, but I learned to change my approach to trying to get as much information from what the patient could describe to me over the phone.” (staff 2)*  *“We were in an old dialysis urology, just a little section downstair on 5G that we were using as a little clinic space. A lot of areas in the hospital had closed down with people working from home, and we had to go to different levels and spread out. There wasn't a real dedicated place for us. We had to find our own space to make all the phone calls and then were kicked out and then had to find another place […] It was so hot … our nurses had to gown up in COVID clothes […]. You had to sit there for hours, […]. You had to leave and doff off to get a drink.” (staff 5)*  *“It's very hard for a health service to provide budget and staffing around a model that is unknown. So as much as it may have seemed like we weren’t listening, there’s also a budgetary concern about committing FTE and resources to something unknown. We didn't know what we were going to need. On reflection of that and on probably lessons learned, it's looking at the base resources that you need to prepare the model efficiently, with a dedicated plan in place for what resources you will use when the model needs to be implemented.” (staff 6)*  **Changing requirements in relation to COVID-19 (barrier)**  *"On one day, you might have a bunch of discharges and no referrals or minimal referrals. One nurse might only have to call five patients, which isn't much... but on other days, you might have to call 30 patients, and it's just hectic." (staff 4)*  *“Admitting all of them like we did from the beginning, every single patient notified were admitted, we then had to go back and cancel all of these admissions after we assessed them because we realised, they didn't need us, or they didn't need that level.” (staff 6)*  *"The amount of administrative resources required to run a service like this were significantly underestimated. There’s a big difference between aiming to avoid hospital presentations for low-acuity COVID-positive patients, and substituting hospital care for those who are acutely unwell with COVID. This distinction is where the administrative workload comes in. We had to quickly create outpatient encounters for all screened patients and then admit only those who required higher-level care." (Staff 7)*  *"We provided remote patient monitoring equipment to many patients, but often gathered results over the phone because the online platform couldn’t handle the quick turnover of numbers we were managing. If we knew we had someone for longer, or we were very concerned and needed to monitor them very closely, we would onboard them to the online platform." (Staff 7)* |
| Maintenance | **Integration to HITH business-as-usual**  *“We still have a lot of patients on our books that have COVID. They will still come to us if they need and we've just included that group of patients into the standard way of how we deliver care.” (staff 7)*  *“We still have the virtual platforms and all that equipment. But that's used for not just COVID-19, but it's also used for a lot of other patients who we might want to monitor their weight daily or their blood pressure.” (staff 5)*  **Workforce capability and skill development**  *“I've learned so many lessons as a leader, as a service manager, as a strategic, project quality improvement, like so much has come out of that, which is really good, even though it was challenging.” (staff 7)*  *“It was a big learning curve. So that if ever faced with that again, we would go, right, we know what to do. Yeah. I think that's been the best thing that's come out of that.” (staff 2)*  *“When people hear about telehealth, there is a fear of the unknown ... Once people realise how easy it is, a lot of that scepticism will be overcome.” (staff 4)*  *“I'm currently now starting a tele-chemo service. So I guess that the experience working in the COVID here, virtual, has really helped in the setup of this new service. I think the time that was spent there was very valuable for me to give another perspective on how treatment can be delivered to patients without them being in here, paying up space in the hospital for other more acute patients.” (staff 4)*  **Potential for scaling to paediatric care**  *“We have a fair few kids that sit on a waitlist for 12 months to get a bowel washout for chronic constipation, which could be done at home … Our diabetes services is getting bigger, but it's still a combined paediatrics adult service. And so they don't have the resources to be able to check on those kids at home” (staff 3)*  **Need for organisational recognition**  *“HITH is very forgotten about sometimes. Maybe people above me need to have a better understanding of what HITH can do. Because at the end of the day, it is cost-saving. It gets people out of hospital. And you know, one of our values is care closer to home.” (staff 3)*  *“I think a lot of the work that HITH does generally is really unseen. And because our patients are at home, it's almost like out of sight, out of mind. Thinking that how much work goes into keeping these patients out of hospital, and the resourcing that's required to maintain and support a model like that, I don't think our team got the right kind of acknowledgement.” (staff 7)* |


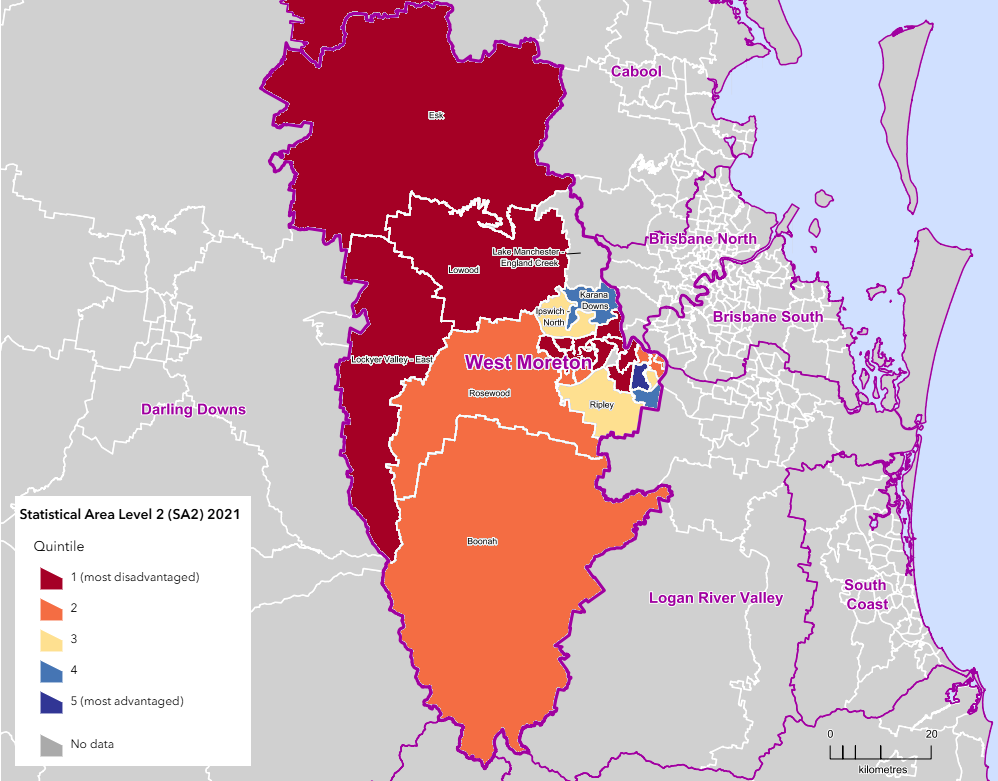
Figure S1: Socioeconomic Distribution of the West Moreton Health Service map by Statistical Area Level 2 (based on ABS SEIFA 2021)

*Source: Australian Bureau of Statistics (ABS). Socio-Economic Indexes for Areas (SEIFA), Australia (latest release 2021); 2023 [Available from* [*https://www.abs.gov.au/statistics/people/people-and-communities/socio-economic-indexes-areas-seifa-australia/2021*](https://www.abs.gov.au/statistics/people/people-and-communities/socio-economic-indexes-areas-seifa-australia/2021)*]*
