# Supplementary material for: Implementation of a Virtual Hospital in the Home Service for Patients With COVID-19 in Queensland, Australia: Mixed Methods Evaluation Using the RE-AIM Framework
Source: J Med Internet Res. 2025 Sep 19;27:e73749. doi: 10.2196/73749 (PMC12495369; doi:10.2196/73749)
Supplement: Multimedia Appendix 2 [file jmir_v27i1e73749_app2.docx]

***‘Tell us about your*** ***healthcare experience’***

***‘Have your say’***

Patient Reported Experience Survey – Inpatient and out of hospital care


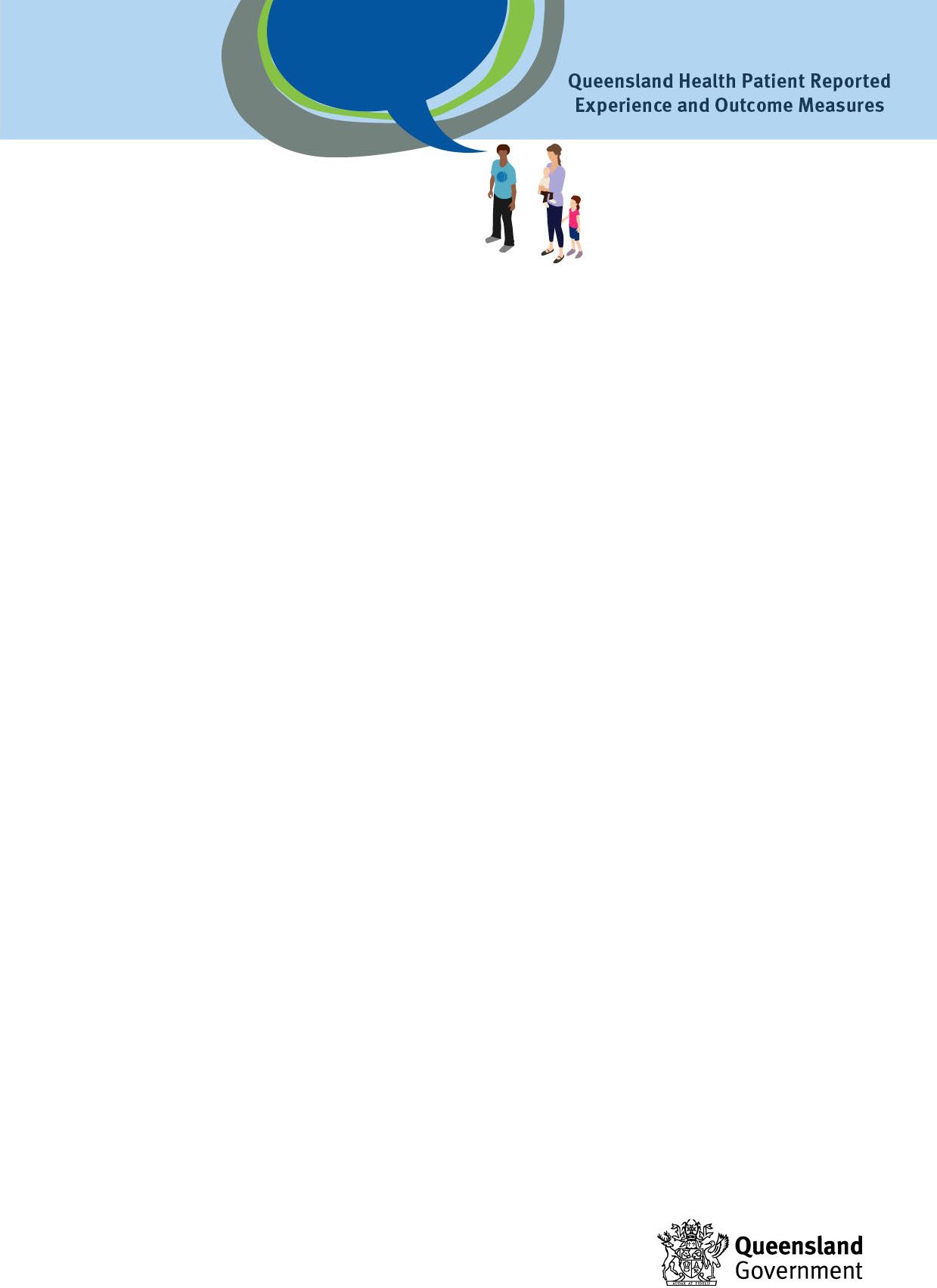

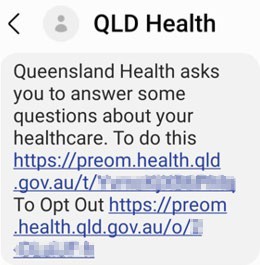


**INPATIENT AND OUT OF HOSPITAL CARE Survey** – Information for patients

***Information for patients who recently received care and treatment in hospital and/or out of hospital***

Queensland Health is asking patients and parents/carers of young patients to take part in an online survey about their experience with the care and treatment received in hospital and/or out of hospital*.* These are known as Patient Reported Experience Measures (PREMs). This feedback will help us find out what we are doing well and what can be improved.

# *Who is included in the survey?*

Most patients will receive an invitation to take part in the survey after they have received care and treatment in hospital or out of hospital. For patients aged less than 18 years, we would like an adult who spent time with the patient to complete the survey.

For mothers who have recently given birth and received care at the hospital, the questions will just be about the care you received.

# *How do I complete the survey?*

A text message (SMS) will be sent to the mobile phone number listed in your/your child’s hospital record **two (2) days after your or your child’s hospital care ends**. The SMS will include a link to the survey. Your phone will need to be connected to the internet to complete the survey.

**Example of the SMS:**

The SMS will include characters at the end of the secure links. These make the links unique for you/your child.

If you have previously provided your email address when completing a different survey, you will be sent an invitation to this survey by email.

After clicking on the survey link, an information page will open, followed by the survey page where you will be invited to take part in the survey.

Version 5.1—October 2022


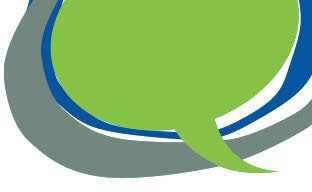


If you agree, you will be invited to answer questions about your/your child’s care that will take about 5 minutes to complete. After this you will be invited to answer additional questions about other parts of your/your child’s care. These will take about 10-15 minutes to complete.

# *Do I have to take part?*

Taking part in the survey is optional. Your/your child’s hospital care will **not** be affected if you choose not to take part. All survey questions are also optional.

# *How do I answer the survey questions if I was in multiple wards during my hospital stay?*

If you/your child stayed in hospital, please think about your experiences in the ward where you/your child received care before leaving hospital when answering the survey questions.

# *What will the survey results be used for?*

We value your feedback as it helps us find out what we are doing well and what can be improved. You/your child will not be identified in the survey results. Your feedback will be combined with others and provided to your hospital. If you include the names of staff, these will not be removed and will be seen by the hospital.

# *Will the hospital monitor my responses?*

Please note individual concerns cannot be responded to. If you have any health concerns, please contact your GP or health care provider. If you or your child have a concern about the care received, please contact the hospital. Your feedback will not affect your/your child’s health care.

If your feedback shows something that may be of serious concern, selected hospital staff will be able to see your/your child’s name. Staff will check the feedback and contact you if needed.

# *Will the information be kept confidential and secure?*

The system used to survey patients is known as Questionnaire Manager and is provided by Philips Electronics Australia Limited.

Survey information is collected for Queensland Health and Hospital and Health Services for the purpose of evaluating, monitoring or planning health services. All information you provide will be securely stored in Australia and handled in line with the *Information Privacy Act 2009* (Qld) and *Hospital and Health Boards Act 2011* (Qld). The information will not be used or shared without your consent, unless allowed or required by law.

For information about how Queensland Health and the hospital protects your/your child’s personal information, visit [www.health.qld.gov.au/global/privacy](https://www.health.qld.gov.au/global/privacy). To learn about rights to access your/your child’s own personal information, visit [www.health.qld.gov.au/system-governance/contact-us/access-info/rti-](https://www.health.qld.gov.au/system-governance/contact-us/access-info/rti-application) [application](https://www.health.qld.gov.au/system-governance/contact-us/access-info/rti-application).

# *What can I do if I do not want to receive a survey?*

If you do not want to receive an SMS inviting you to take part in a survey, or do not want your/your child’s name and contact information to be provided to Philips, contact 13 HEALTH (13 43 25 84).

# *How can I make a compliment or complaint about the health service?*


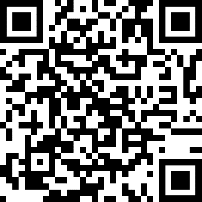
The survey is not the way to make a complaint. If you would like to share a compliment or make a complaint about the health service, information on how to do this is available at [www.qld.gov.au/health/contacts/complaints](https://www.qld.gov.au/health/contacts/complaints).

# *Where can I find more information?*

For more information about Queensland Health Patient Reported Experience and Outcome Measures visit [www.health.qld.gov.au/preom](http://www.health.qld.gov.au/preom) or scan the QR code.

For help to complete a survey contact 13 HEALTH (13 43 25 84).

Information for patients—PREMs Inpatient survey 2
