# Supplementary material for: Implementation of a Virtual Hospital in the Home Service for Patients With COVID-19 in Queensland, Australia: Mixed Methods Evaluation Using the RE-AIM Framework
Source: J Med Internet Res. 2025 Sep 19;27:e73749. doi: 10.2196/73749 (PMC12495369; doi:10.2196/73749)
Supplement: Multimedia Appendix 3 [file jmir_v27i1e73749_app3.docx]

Multimedia Appendix 3 – Interview guide

Staff

**Q1. Can you please tell me a bit about your role? What does a typical day in your job look like?**

- How has your role evolved over time, especially in relation to Hospital in the Home (HITH)?
- What led you to join the HITH service?

**Q2. Can you please provide some insights about HITH service?**

- How was the service set up?
- Any main factors contributing to the decision of taking up the service?
- When designing, what key stakeholders were on board?
- Whose work or workflows could potentially be impacted by this implementation?

**Q3. Was there any changes or adaptations that were made to the service since its initial implementation, and why these changes were made?**

- Are there any guidelines from Queensland Health you need to follow?
- Did you deliver the service adherence to any standards of care?
- What happened after the service was decommissioned? Is telehealth still being used for HITH?

**Q4. How do you feel about the setup and design of this service?**

- Which aspects are good/ not good?

**Q5. Can you please let me know how has delivering services through HITH has impacted your day-to-day activities? Are there any specific aspects that stands out?**

- Do you have any concerns before and after the service was implemented? Is it very different from working for a traditional physical ward?
- Do you receive any supports, or having difficulties when delivering the service?

**Q6.1 Can you please identify which factors have supported you to successfully deliver the service?**

- Any specific skills required? How would you get them?
- How could WMH support you to get them?
- Equipment, technology, funding, improved referral pathways, organization support

**Q6.2 And what were the barriers to delivering this service?**

**Q7. What are your thoughts on the safety and effectiveness of HITH when comparing it with a traditional hospital bed?**

- Can you share any specific examples or experiences that influenced your perception?

**Q8.1 What are some of the benefits that the HITH model has brought to patients from your opinion?**

- received the right care at the right time, feel at ease being in your own home/ surroundings, not having to arrange care for children/ dependants.

**Q8.2 And how about the negative aspects?**

- the set-up process too confusing, problem with the technology, concerns about privacy/ the security of your health information.

**Q9. How would you assess WMH’s HITH service compared to other HHS?**

- Which aspects are good/ not good?

**Q10. Do you have any suggestions for how this HITH model of care could be improved in the future?**

- Are there any specific changes you would suggest in the context of caring for COVID-19 patients?
- Which sorts of clinical areas, and in which situation do you think that the HITH model of care is most appropriate, apart from COVID-19?

Patient

**Q1. Please tell me how acceptable was using the virtual COVID-19 HITH services for you?**

**Q2. How did you become aware of the WMH virtual COVID-19 HITH service?**

**Q3. Why do you think you need support from a professional COVID-19 healthcare service?**

**Q4. What happened during your admission to the service?**

- How was the health professionals’ attitude?

**Q5. During your admission, what did you find the most valuable about the service to help you manage your COVID-19 symptoms at home?**

- Was the timing of each consultation enough to discuss your medical problem?
- Did the information given helped with your worries or fears?
- Were your view/ cultural or religious beliefs respected?
- Were you given enough support to feel at ease being at home?

**Q7. Thinking about your experiences of receiving care remotely through phone call and remote monitoring devices, were there any aspects that you felt annoying compared to being in a regular ward and receiving care face-to-face?**

- Did you have any problems with the set up process or technology?
- Do you have any concerns about privacy/ the security of your health information?

**Q6. Did you have to pay any out-of-pocket costs associated with your admission?**

**Q7. How likely would you be to recommend the service to your friends or family member who was in your situation with COVID-19?**

**Q8. In what situations would you be happy to be admitted to a virtual ward instead of a regular hospital ward? And in what situations would you not be happy to use?**

**Q9. Any final feedback? Is there anything you feel that the virtual COVID-19 HITH service could have done better?**

**DEMOGRAPHIC INFORMATION**

1. How old are you?
2. How do you describe your gender?
3. What is the highest level of education you have completed?
4. Are you of Aboriginal or Torres Strait Islander descent?
5. What country were you born?
6. What is the main language you speak at home?
