# Supplementary material for: Implementation of a Virtual Hospital in the Home Service for Patients With COVID-19 in Queensland, Australia: Mixed Methods Evaluation Using the RE-AIM Framework
Source: J Med Internet Res. 2025 Sep 19;27:e73749. doi: 10.2196/73749 (PMC12495369; doi:10.2196/73749)
Supplement: Multimedia Appendix 4 [file jmir_v27i1e73749_app4.docx]

#

**Model of Care**

Virtual COVID-19 Hospital in the Home Service planning

December 2021

**CONTENTS**

[CONTENTS 2](#_Toc169625450)

[ABBREVIATION LIST 3](#_Toc169625451)

[1. Introduction 4](#_Toc169625452)

[1.1 Overview 4](#_Toc169625453)

[1.2 Model principles 5](#_Toc169625454)

[1. 3 Objectives 6](#_Toc169625455)

[2. Model description 7](#_Toc169625456)

[2.1 Sources of COVID-19 patients 8](#_Toc169625457)

[2.2 Patient care pathways 10](#_Toc169625458)

[2.2 Admission process 12](#_Toc169625459)

[2.2 Medical Governance 13](#_Toc169625460)

[2.3 Staffing profile 15](#_Toc169625461)

[2.4 After hours workflow 16](#_Toc169625462)

[2.5 Facilities requirements 16](#_Toc169625463)

[2.7 Record keeping 17](#_Toc169625464)

[2.9 Discharge from a care stream 17](#_Toc169625465)

[3. Service governance 18](#_Toc169625466)

[3.1 Catchment Area 18](#_Toc169625467)

[3.2 Other supports 18](#_Toc169625468)

[3.3 Costing 18](#_Toc169625469)

[Appendix 1 19](#_Toc169625470)

[Appendix 2 23](#_Toc169625471)

[Appendix 3 25](#_Toc169625472)

[Appendix 4 26](#_Toc169625473)

[Appendix 5 27](#_Toc169625474)

[Appendix 6 28](#_Toc169625475)

# ABBREVIATION LIST

| COVID-19 | Coronavirus disease of 2019 |
| --- | --- |
| HITH | Hospital in the home |
| HHS | Hospital and health service |
| WMH | West Moreton Health |
| PHN | Primary Health Network |
| GP | General practitioner |
| QAS | Queensland Ambulance Service |
| QCH | Queensland Children Hospital |
| CNC | Clinical Nurse Consultant |
| ieMR | Integrated electronic medical record |

1. Introduction

## 1.1 Overview

This document outlines the clinical and operational requirements of the West Moreton Health (WMH) Virtual COVID-19 Hospital in the Home (HITH) Model. We aim to provide support the community due to Queensland transitions to pandemic mode. The WMH Virtual COVID-19 HITH Model is intended to be dynamic to adapt to changes required FOR keepING the community safe. The Model will also continually be reviewed, tested via desktop exercises, adjusted as directives from the state/Commonwealth are updated, and/or the situation continues to develop.

In the case of an outbreak in Queensland, the current epidemiological modelling of a COVID-19 outbreak predicts significant demand and hospital systems will be overwhelmed consuming all available resources.

At a state-wide level the following services have been engaged, Office of the Chief Health Officer, Hospital and Health Service Chief Operating Officers, Public Health Unit, Primary Healthcare Networks, Aboriginal and Torres Strait Islander Community Controlled Health Organisations and several Queensland Health departments have been working to develop a comprehensive model to address this demand.

West Moreton Health will provide a ‘whole family’ model enabling parents and children to be supported through the one service. This will alleviate unnecessary stress on families during already difficult times.

**Figure 1: Queensland Health Corporate Governance**

## 1.2 Model principles

West Moreton Health remains a ‘Spoke’ with Metro South Hospital and Health Service being our ‘Hub’ for adults as well as connection to the Queensland Childrens Hospital virtual care program for paediatrics. The following principles support the hub and spoke model.

The Hub and Spoke relationship aims to facilitate and maximise the ability of the health system to provide high quality care for people as close to home as can be safely achieved. It utilises the resourcing, clinical experience, and available labour of the Hub to support the specific knowledge of the person and the local environment of the Spoke to maximise efficient, person centric care. The principles for the Hub/ Spoke relationship are below:

**Principle 1**

The majority of virtual care will occur at the Spoke. The Spoke is best placed to provide care for the person close to home. Understanding the local resource environment, having strong relationships with the local primary care resources and the ability to facilitate timely safe escalations and de-escalations.

**Principle 2**

Escalation and de-escalation and clinical advice are coordinate through the medical officer of the Spoke virtual ward supported by the medical officer governing the Hub when required. Discussion with Metro South would need to occur as a whole workflow needs developing.

**Principle 3**

The Hubs role is to provide support to the Spoke as needed operationally and clinically. This support can include clinical advice, operational advice, virtual monitoring, or labour support.

**Principle 4**

Clear communication processes / channels between Hub and Spoke are required with visibility of all care notes available for Hub/Spoke access.

**Principle 5**

The normal escalation path from the Spoke will be to the Hub for both clinical advice and physicalised escalation of the person.

**Principle 6**

The Spoke shall maintain its business as usual for as long as it can with the support of the Hub until the Hubs capacity is exhausted or finalised. Decreases to normal clinical are should only occur after the Hub support has been maximised.

**Principle 7**

The relationship between the Hub and Spoke should be based on shared data, shared data access and transfer of care through direct clinical contact.

**Principle 8**

The Hub and Spoke should develop their relationships with each other and their local primary care networks before there is significant strain on the system due to increasing numbers of people with COVID-19.

**Principle 9**

The Primary Health Network (PHN) General Practitioners should be involved with the design of community COVID-19 care beginning at the HHS level.

**Principle 10**

Pathway for escalations/de-escalation between COVID well, COVID of concern and Hospital should be established with a single point of contact established for WMH.

**Principle 11**

Peoples choices are fundamental but where appropriate and acceptable to the persons involved, households should be treated as units to minimise disruption and redundant communication to the household

## 3 Objectives

The objectives of the Model are to:

- Provide operational guidance, with a focus on actions and responses to support COVID-19 patients.
- Minimise transmissibility, morbidity and mortality for people diagnosed with COVID-19.
- Provide an alternative to traditional hospital care in the community setting for patients of all ages.
- Reducing unnecessary burden on the inpatient health care setting.

**Figure 2: West Moreton Health virtual COVID-19 HITH service objectives**


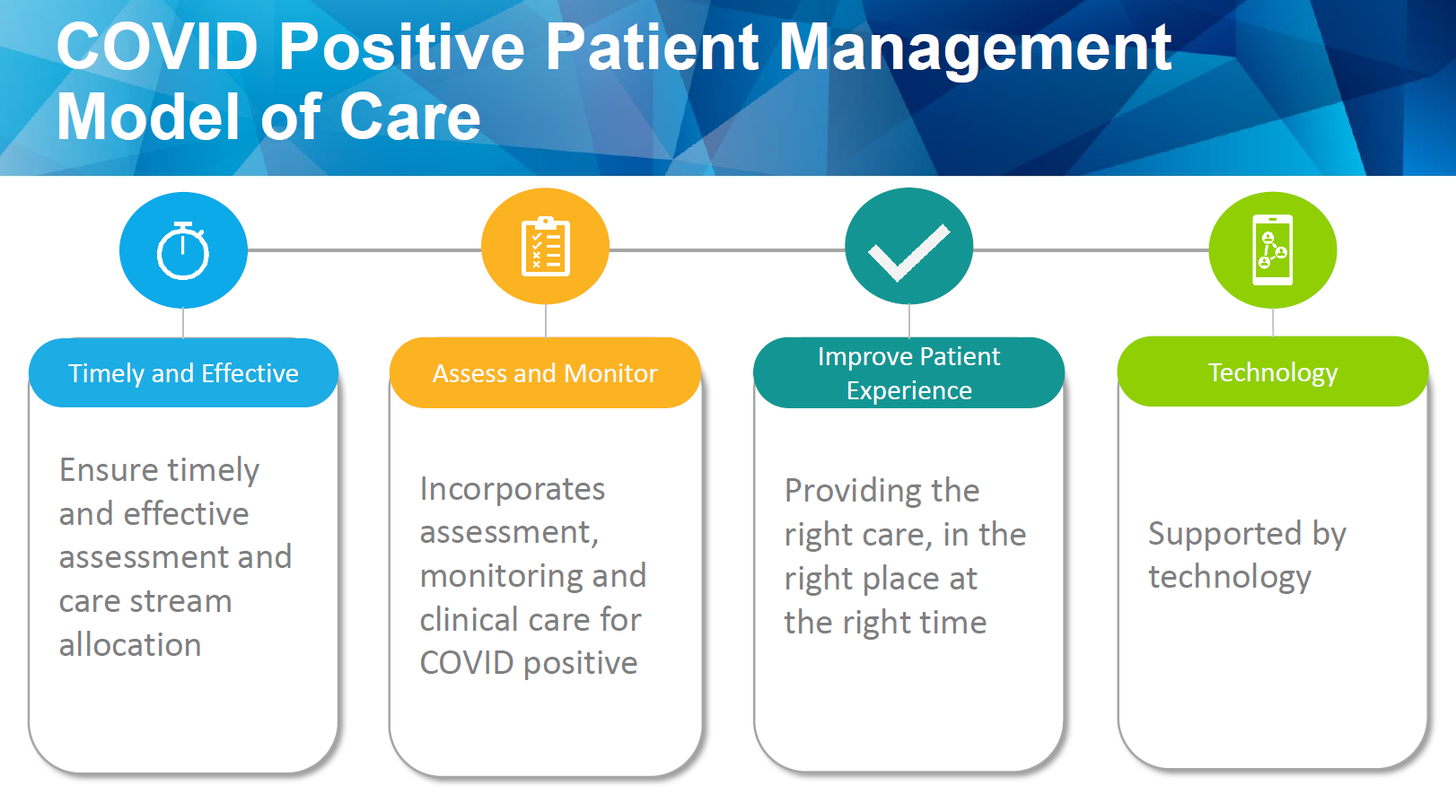


# Model description

West Moreton Health Virtual COVID-19 HITH Model aligns with the implementation of the health direct platform at a state level. Once patient return positive results, there are three proposed pathways for each patient as listed in figure 3.

**Figure 3: Three clinical care streams for COVID-19 positive patients**


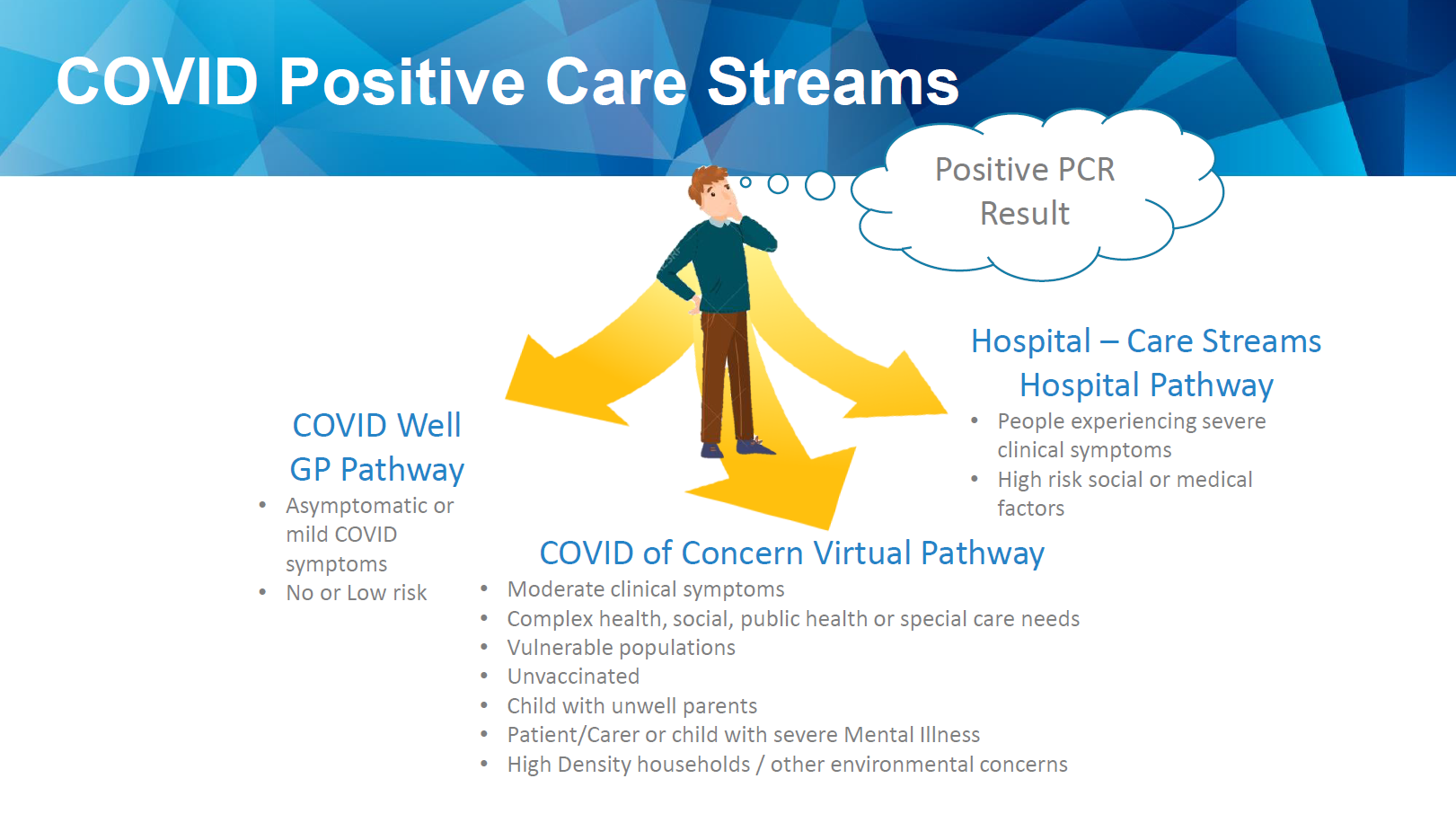


## 2.1 Sources of COVID-19 patients

**Healthdirect Australia Screening**

Healthdirect Australia will undertake initial contact and screening of people through a telephone interview within six hours of being notified of a positive COVID-19 PCR result. This survey will identify individuals of concern and allocate and refer people to the appropriate care stream. The initial screening will:

- Identify the person
- Confirm their identity
- Collect key demographic information
- Confirm their geographical location
- Confirm the capacity of the person
- Confirm consumer consent and identification process
- Consent the person for the provision of community care, the collection and transfer of data and financial consent
- Provide details of a web page with existing processes for social services
- Identify public health concerns and transfer data to NoCS for public health unit management
- Identify clinical concerns and immediately escalate to nominated HHS delegate to determine allocation to Hospital or COVID of concern care stream
- Refer prioritised list of people assessed as COVID of concern to HHSs for further assessment and management
- Allocate people identified through the initial screening process to be low risk (see Care streams) to the COVID well care stream and refer to a governing general practitioner (GP), GP respiratory clinics or Aboriginal and Torres Strait Islander Community Controlled Health Organisations (ATSICCHOs)

**General Practitioner Care**

GPs will provide the governance and clinical care of the majority of people with COVID-19 (COVID well care stream). This care is supported by the [RACGP guidelines](https://www.racgp.org.au/clinical-resources/covid-19-resources) and the local HHS health pathway website COVID-19 suite. A list of GPs for the WMH catchment will be provided by the Primary Health Network (PHW).

**Hospital and Health Service Screening**

WMH will undertake a detailed admission screening process to risk stratify COVID-19 positive people and determine management needs. People allocated to the COVID of concern care stream will be monitored by WMH throughout their infectious period and have a final assessment prior to discharge.

If required, WMH can establish a Hub and Spoke relationship to escalate additional support requirements. Where initial contact is via WMH instead of Healthdirect Australia, the same screening and actions as noted in the “Healthdirect Australia Screening” section are required to be completed.

**
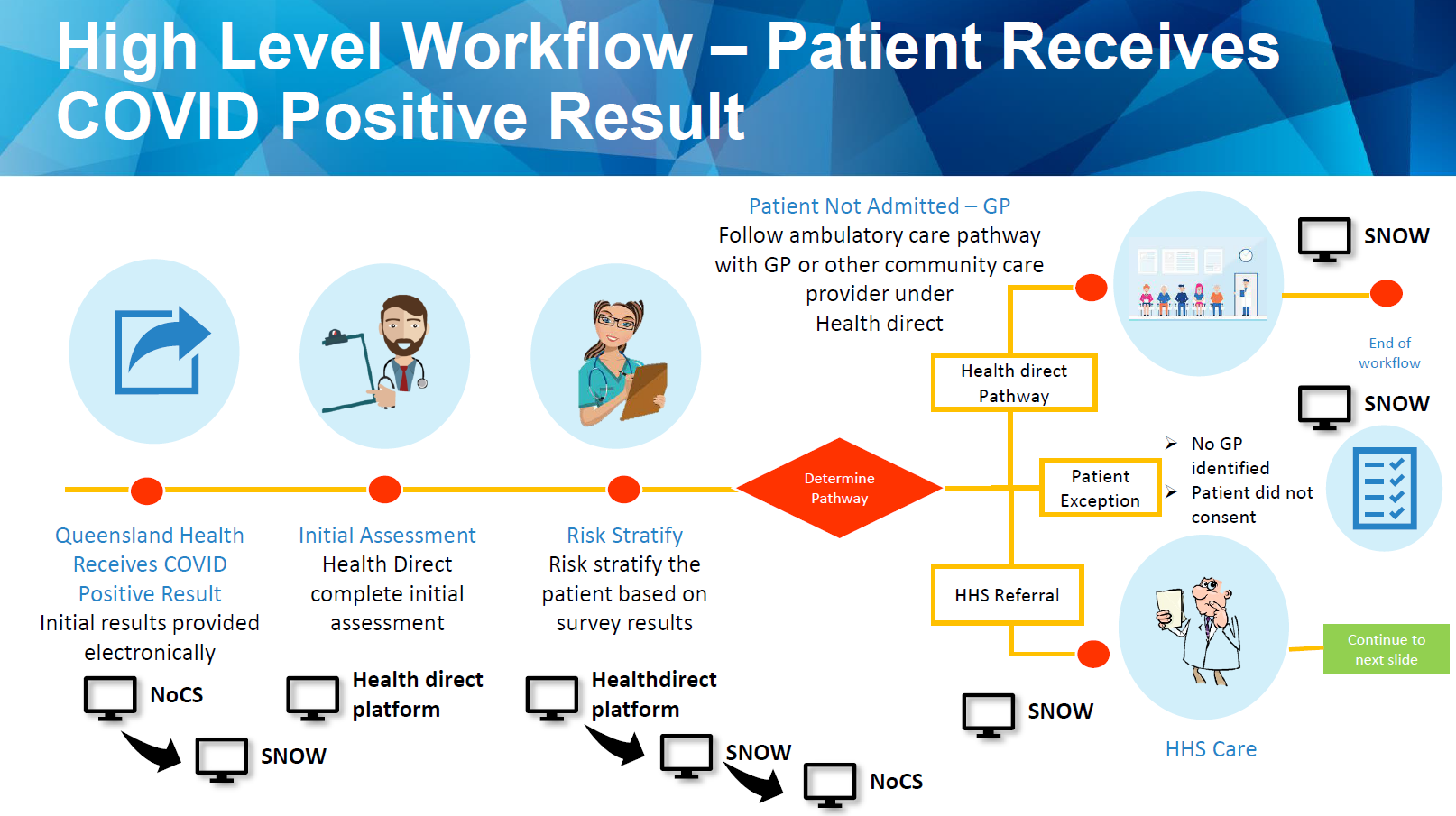
Figure 4:** **West Moreton Health virtual COVID-19 HITH service - High Level Workflow**


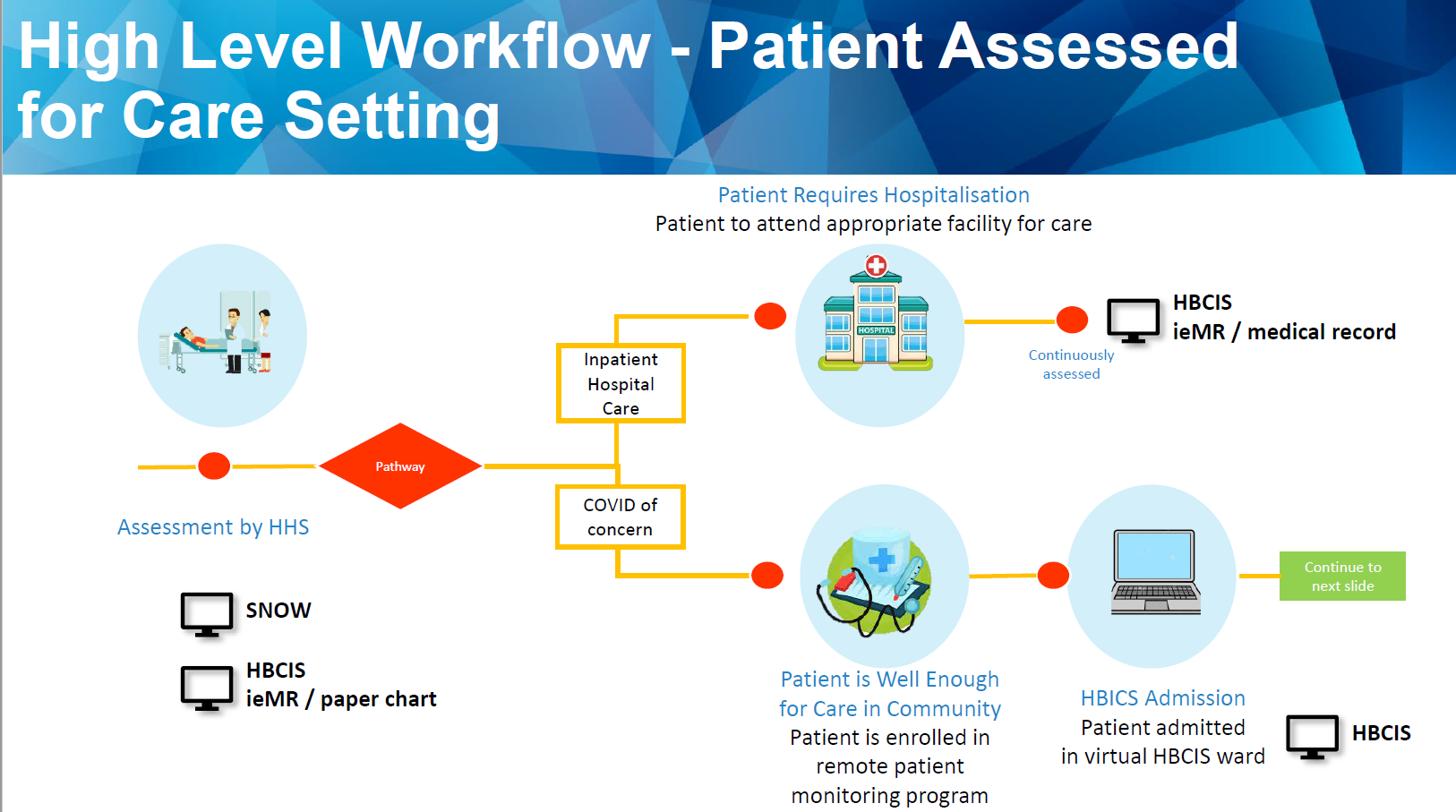


## 2.2 Patient care pathways

The Virtual COVID-19 HITH team will operate in the clinic from 08:00 – 16:30 7 days per week, providing rapid assessments for COVID-19 positive patients (within business hours). The telehealth hub will operate 24 hours per day 7 days per week, including public holidays.

Escalation pathways for the clinical unwell patient will be to a designated COVID-19 Hospital (either Ipswich Hospital, PA Hospital or Queensland Children Hospital) depending on the level of clinical deterioration. Queensland Ambulance Service (QAS) will also determine the pathway for the patient to the closest Emergency Department depending on the pateints clinical condition. When a flag is raised within the virtual platform, an initial assessment via telephone will be conducted to determine ongoing care requirements. This undertaken where a clinical determination/decision regarding the most appropriate pathway for the patient will be provided. Pathways are listed below:

**Figure 5: West Moreton Health virtual COVID-19 HITH service - Adult and Maternity Patients care pathways**


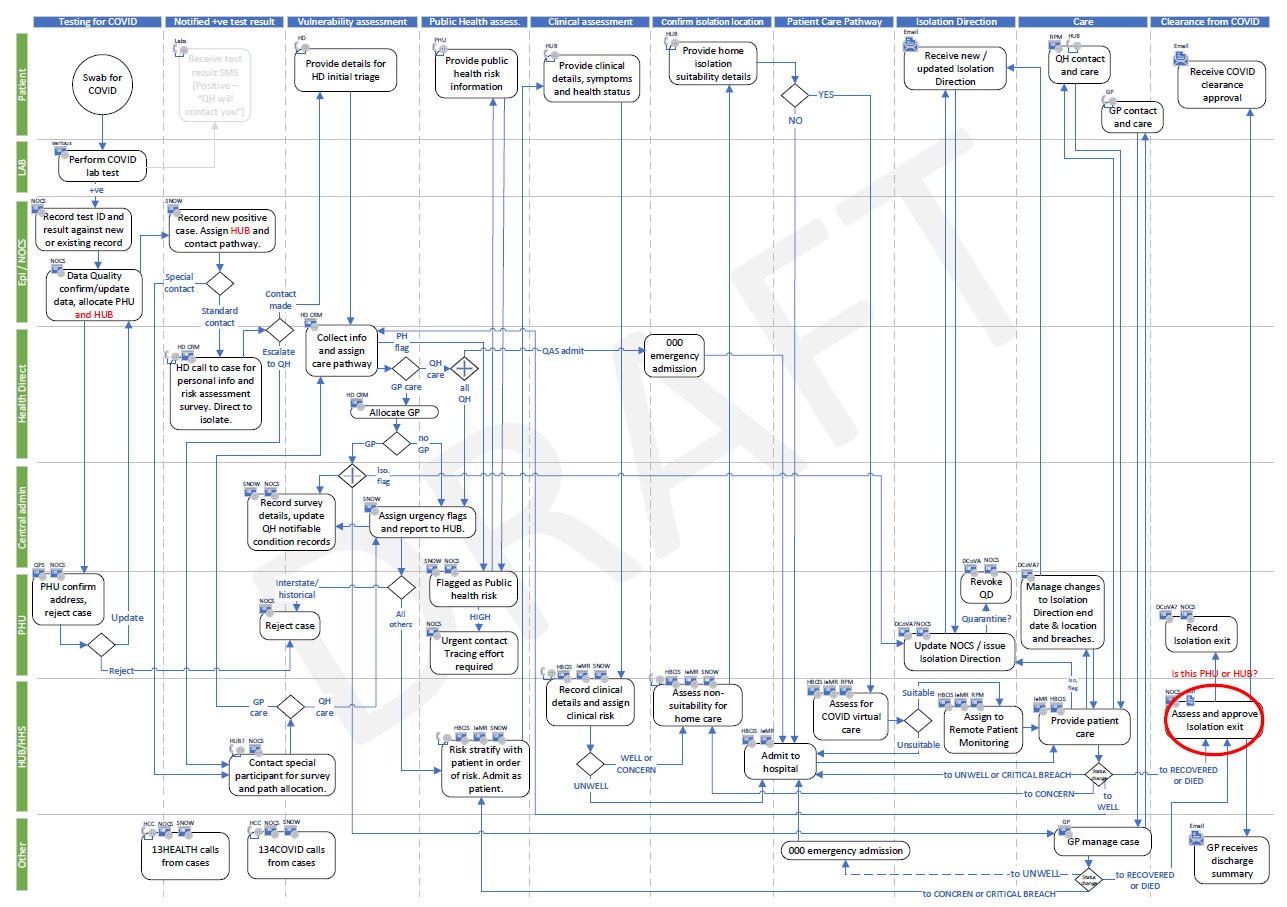


**Paediatric Patients**

Paediatric patients enrolled in the Virtual COVID program will following the below management plan developed by Childrens Health Queensland. Coordination of transfer would occur with Queensland Children Hospital (QCH) via the Disaster and Emergency Incident Plan determine bed availability.

**Figure 6: West Moreton Health virtual COVID-19 HITH service - Paediatric Patients care pathways**


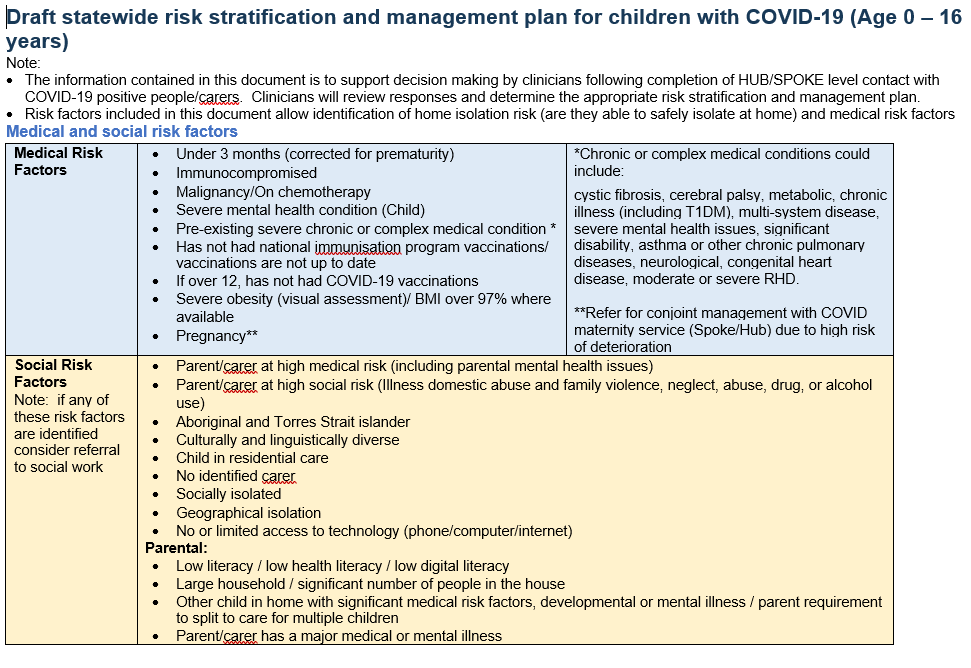


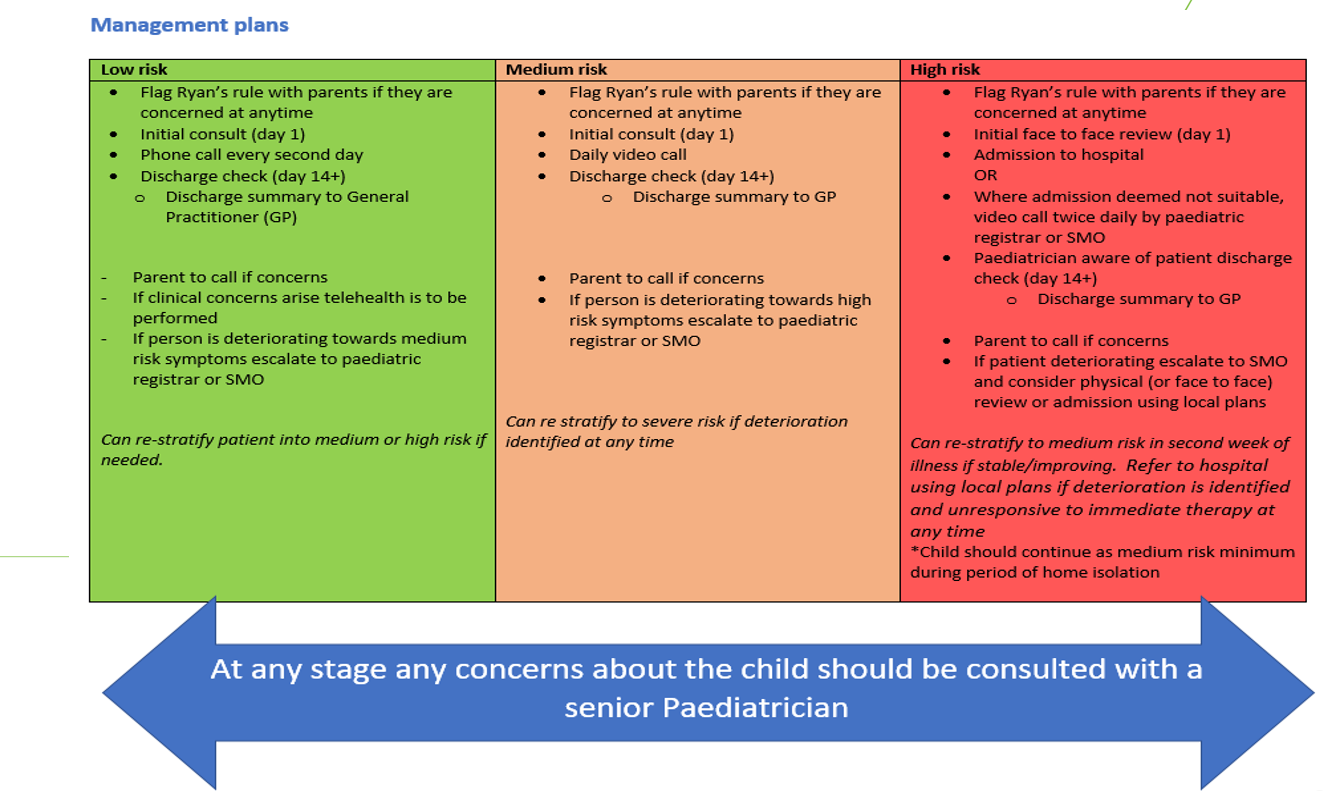


## 2.2 Admission process

**Figure 7: West Moreton Health virtual COVID-19 HITH service – Admission process**

## 2.2 Medical Governance

Strong medical governance is the underpinning principle of safe patient care. To ensure that the Virtual COVID-19 HITH Model keeps patients safe, the below diagram outlines who maintains medical governance of the patient during what episode of care.

**Figure 8: West Moreton Health virtual COVID-19 HITH service – Medical governance**

**Governance remains with Virtual COVID Consultant**

**Governance remains with Virtual COVID Consultant**

**COVID Positive Patient**

**Virtual COVID Platform**

**Clinic Assessment**

**Telephone support***

**Governance remains with Virtual COVID Consultant**

(*Telephone services include telehealth and telephone follow-up)

Appendix 1 provides a COVID-19 readiness self-assessment to determine preparedness for managing people with COVID-19.

Care for patients 16 years old or older will be provided by the Virtual COVID-19 HITH Clinical Consultant.

All patients 16 years and under will receive medical governance fromthe paediatrician. A pathway will exist for the Clinical Consultant to escalate any concerns directly to the Paediatrician when received from the clinical team for review and intervention by the Paediatric team. Links to the QCH Virtual COVID-19 program are established as a point of escalation for the medium to high risk paediatric patient.

## 2.3 Staffing profile

The table below outlines the initial workforce profile, this will need scaling as cases increase.

The proposed staffing profile could support up to 50 COVID-19 positive patients noting that the clinic would support 12 face to face reviews per day. Per additional 50 patients the below staffing would be required.

**Table 1: West Moreton Health virtual COVID-19 HITH service – Staffing profile**

| Role | Responsibilities |
| --- | --- |
| Medical Consultant (Lead) – Adults (1.0 FTE)  On call service available on weekends and afterhours | - Providing review on adult patients (based criteria) in Virtual Platform - Governance and medical advice for service - Acute assessments of COVID-19 positive patients in clinic setting - Telehealth review – Remote hospital inpatients - Education for other medical and nursing staff on providing holistic care to adult patients with COVID-19 |
| Medical Consultant – Paediatrics (Consult Service only) | - Providing review on paediatric patients (based criteria) in Virtual Platform - Governance and medical advice for service including after hours and on weekends - Acute assessments of COVID-19positive paediatric patients in clinic setting - Education for other medical and nursing staff on providing holistic care to adult patients with COVID-19 |
| Medical Registrar (1.0FTE) Adults  Working weekends supporting clinic reviews | - Telehealth review for patients of concern (based on Flags on MyCareManager system) - Acute assessments of COVID-19 positive patients in clinic setting - Ward work – prescriptions, medical documentation, discharge summaries, preparing criteria led discharges (with consultant oversight) |
| Resident medical officer (1.0 FTE) | - Telehealth review for patients of concern (based on Low acuity Flags on MyCareManager system) - Ward work – prescriptions, medical documentation, discharge summaries, preparing criteria led discharges (with consultant oversight) - Medical cover for Sotrovimab infusions |
| Clinical Nurse Consultant (1.0 FTE) Mon – Fri  Adults | - Senior nurse for model - Patient reviews via virtual platform and in clinic setting - Service management (HR, Payroll, Rostering, Quality Improvement, Recruitment, data reporting, financial management) |
| Clinical Nurse Consultant   1. FTE) Mon – Fri   Peadrictics | - Senior nurse for model - Patient reviews via virtual platform and in clinic setting - Service management (HR, Payroll, Rostering, Quality Improvement, Recruitment, data reporting, financial management) |
| Clinical Nurse Midwife  (0.4) Mon – Fri | - Follow-up phone calls - Monitoring patients remotely via virtual platform - Clinic based patient reviews - Team leader in the absence of CNC - Staff allocated between clinic and telehealth hub - ED skill set ideal given broad range of skills for all age groups |
| Clinical Nurses (4.41 FTE)  3 x Shifts 7 days  (1 AM, 1 PM, 1ND) | - Follow-up phone calls - Monitoring patients remotely via virtual platform - Clinic based patient reviews - Team leader in the absence of CNC - Staff allocated between clinic and telehealth hub - ED skill set ideal given broad range of skills for all age groups |
| Registered Nurses (11.76 FTE)  8 x Shifts 7 days  (4 AM, 4 PM) | - Monitoring patients remotely via virtual platform - Clinic based patient reviews - Staff allocated between clinic and telehealth hub - ED skill set ideal given broad range of skills for all age groups - Donning and Doffing of PPE spotter role |
| Operational Officers (2.8 FTE) 2 shifts 7 days  1 AM, 1 PM | - Cleaning clinic after use - Assisting in assessments |
| Administration Officer (2.8 FTE) 2 shifts 7 days  1 AM, 1 PM | - Data entry to ESM and other data systems - General office duties (stationary ordering etc) |
| Pharmacy HP3  (1.0FTE) M-F | - Supply of medication to patients on Virtual program - Medication reconciliation and ELMS on discharge |

## 2.4 After hours workflow

The current Virtual COVID-19 HITH Model supports 24/7 nursing staffing with on call medical support. Patients and GPs will be able to speak directly to a senior nurse for support and after hours accommodation for the role will be within the Patient Access and Coordination Hub (PACH) Ipswich Hospital, Level 7. New referrals will not be accepted to the program after hours.

## 2.5 Facilities requirements

**Digital Equipment**

The model will require a main phone line into the service with the ability for this system to ensure each call is answered (phone hunt). This number will be provided to patients, QAS, GPs and PA Hospital which will be WMH’s Virtual COVID-19 HITH Hub.

The Digital hub requires:

- 10 Laptop computers, with docking stations, keyboards, mouse, and dual monitors
- 10 desk phones with headsets

The clinic requires:

- Pathology equipment
- Basic observational equipment
- Resus trolley including defib
- PPE for caring for positive COVID patients
- Medical imaging equipment
- Repurposed Workstations on Wheels from current fever clinic area

**Accommodation**

The virtual COVID-19 HITH model requires a collocation base. The current fever clinic in Court Street is a suitable location for this model to operate as rapid assessment of the COVID positive patient can be completed in a safe manner with access to portable Xray services and pathology in this space. Medical imaging will conduct a review of this location to ensure it is suitable from a radiology perspective and a mobile x-ray unit will remain onsite:

Room requirements as below:

- 3 x clinic rooms
- 1 x room suitable for Xray
- 1 x reception/office space for admin staff
- Donning and doffing areas
- Staff breakout area for breaks

A digital hub for staff to work remotely with the following requirements:

- dashboards outlining current activity mounted in clearly visible areas to the team
- 2 x offices (can be shared) – (1 for CNC & 1 for consultant)
- 9 desks with telephones and computers

Notes: depending on the number of positive COVID-19 patients these areas may need to be scaled if the situation was to rapidly increase. This could include a rural location for clinic assessments. Additional space in the digital hub will also be required

## 2.7 Record keeping

Contact record keeping of people allocated to the COVID well or COVID of concern care stream will be on the Queensland Health central data platform.

For this WMH virtual COVID-19 HITH model, patients’ monitoring details, contact notes, observations and clinical actions is generated as a pdf file and transferred to relevant local system at the conclusion of the episode of care. The integrated electronic medical record (ieMR) local system will be the clinical record of the episode of care. Clinical records are completed as per usual HHS record keeping processes. An electronic summary of care provided to date must be provided each time a person moves between the three care streams to allow timely, safe continuous care.

Where WMH provides care related to a COVID-19 positive PCR result, the person should be admitted under one of the following codes:

- VCOV01 - for people being managed in a virtual care model
- HCOV/HOMECO – for people with COVID-19 being managed in a standard HITH environment in accordance with the Queensland HITH guideline. HHSs who have an existing HOMECO ward can use the existing code to admit people to the HITH virtual ward. If a new ward to care for people with COVID-19 in a HITH model needs to be established, the HCOV code should be used.

Note: Sites can append a numeric or alpha character after HCOVxx or VCOVxx.

## 2.9 Discharge from a care stream

Discharge from a care stream will occur when the person has met the criteria for release from home isolation directions as per the national COVID-19 guideline and are determined to have no further acute care needed. Utilising the PHN end of episode template, GP removes person from COVID well pathway and informs Queensland Health.

WMH medical officer providing clinical governance for COVID of concern or Hospital care streams, discharges person after clinical review (ensuring national guideline requirements are met) and informs PHN. PHN may require additional information for closure of case in National office for Child Safety

Once a person is discharged from WMH, a discharge summary must be completed, uploaded to The Viewer within 24 hours, and update the central data platform.

# Service governance

## 3.1 Catchment Area

The Virtual COVID-19 HITH Model services all patients in the West Moreton Health Service area. Clinic location will initially be Ipswich CBD, however if required a rural location maybe considered to provide the option of care closer to home and reduce travel for positive COVID-19 patients.

## 3.2 Other supports

It is acknowledged that patients receiving care from this WMH virtual COVID-19 team will require input from other disciplines throughout their care journey and care/consult may be needed to be redirected from services to provide dedicated care for the program

Existing internal pathways for referral already exist through the smart referrals (WM-Refer) platform. Patients that require ongoing support will be referred through this mechanism.

Additional care providers include:

- Allied Health (noting some disciplines may be needed in the scale of the model, eg physio)
- Mental Health services – Adult, CYMHS, Older persons
- AODS support
- Medical/Surgical Sub Specialist
- Queensland Ambulance Serivce
- Publih Health Team
- Pharmacy
- Other non COVID related requirements eg, transport assistance.

## 3.3 Costing

**Table 2: West Moreton Health virtual COVID-19 HITH service – Labour cost**

| **Costing** | **Stream** | **Grade** | **Paypoint** | **Employ Status** | **Start Date** | **End Date** | **FTE 2021-22** | **Headcount 2021-22** | **FY Total** | **Comments** |
| --- | --- | --- | --- | --- | --- | --- | --- | --- | --- | --- |
| 1 | Senior Medical Officers | L27 | 20MMOI2 03 | Permanent | 1/07/2021 | 30/06/2022 | 1 | 1 | 530199.74 | Mon-Fri, oncall & O/T |
| 2 | Resident Medical Officers | L13 | 20MSREG 04 | Permanent | 1/07/2021 | 30/06/2022 | 1.47 | 1 | 368552.09 | 7 Days |
| 3 | Resident Medical Officers | L8 | 20MEDREG 05 | Permanent | 1/07/2021 | 30/06/2022 | 1.47 | 1 | 297468.2 | 7 Days |
| 4 | Nurses & Midwives | NRG7 | 30NRG7 04 | Permanent | 1/07/2021 | 30/06/2022 | 1 | 1 | 170113.48 | Mon-Fri |
| 5 | Nurses & Midwives | NRG6 | 30NRG6-1 04 | Permanent | 1/07/2021 | 30/06/2022 | 1.47 | 2 | 231951.24 | 7 Days AM shift |
| 6 | Nurses & Midwives | NRG5 | 30NRG5 04 | Permanent | 1/07/2021 | 30/06/2022 | 5.89 | 6 | 760284.81 | 7 Days AM shift |
| 7 | Nurses & Midwives | NRG1 | 30NRG1 04 | Permanent | 1/07/2021 | 30/06/2022 | 2.94 | 3 | 275722.52 | 7 Days AM shift |
| 8 | Administrative | AO3 | 10AO3 04 | Permanent | 1/07/2021 | 30/06/2022 | 2.94 | 3 | 331029.59 | 7 Days AM shift |
| 9 | Nurses & Midwives | NRG6 | 30NRG6-1 04 | Permanent | 1/07/2021 | 30/06/2022 | 1.47 | 2 | 239828.83 | 7 Days AM shift |
| 10 | Nurses & Midwives | NRG6 | 30NRG6-1 04 | Permanent | 1/07/2021 | 30/06/2022 | 1.47 | 2 | 249445.52 | 7 Days ND shift |
| 11 | Nurses & Midwives | NRG5 | 30NRG5 04 | Permanent | 1/07/2021 | 30/06/2022 | 5.89 | 6 | 786067.78 | 7 Days PM shift |
| 12 | Health Practitioners | HP4 | 75HP4 04 | Permanent | 1/07/2021 | 30/06/2022 | 1.47 | 2 | 270993.91 | 7 Days AM shift |
|  | **Total** |  |  |  |  |  |  |  | **4,511,657.72** |  |

Appendix 1

**Table 3: Hospital and Health Service COVID-19 readiness self-assessment**

This checklist provides assurance that Hospital and Health Services:

1. will be prepared to cope at peak demand
2. have identified the risks to their provision of care to COVID-19 positive persons within their HHS
3. have outlined mitigation strategies against these risks
4. have established clear governance of their virtual care model

| **1** | **Capacity to respond to demand** | | | |
| --- | --- | --- | --- | --- |
| 1.1 | Have you identified the capacity of your virtual care model (initial and at peak) | Base volume | Expanded volume | Comment |
| 1.2 | Have you identified the capacity of your Hospital model (initial and at peak) | Base volume | Expanded volume | Comment |
| 1.3 | Have you identified the capacity of your Hospital in the Home model (initial and at peak) | Base volume | Expanded volume | Comment |
| 1.4 | Do you have capacity to cope with the expected volume of patients predicted at peak | Yes/No | Mitigation strategies (engage Hub for support) | |
| 1.5 | Have you reviewed available local primary care to identify gaps in primary care to support community virtual care | Yes/No | Mitigation strategy | |
| 1.6 | Do you have a surge workforce plan? | Yes/No | If no, how are you planning to manage this | |
| **2** | **Virtual care model** | | | |
| 2.1 | Is there a dedicated clinical staff member be contacted 24/7 for your virtual COVID model? | Yes/No | Who is delivering after hours support for you virtual model of care? | |
| 2.2 | What is the dedicated number for this service |  | | |
| 2.3 | Do you have capacity to respond to referrals from Healthdirect Australia **within 12 to 24 hours?** | Yes/No | If no, how are you going to mitigate this | |
| 2.4 | Do you have a dedicated number for Healthdirect Australia to transfer patients directly to a clinician between 0700hrs and 2200hrs? | Yes/No | If yes, what is the plan if this number is busy | |
| 2.5 | Is after hours clinical capacity in place to monitor and respond to patients overnight? | Yes/No | If no, what is the plan for patients overnight | |
| 2.6 | Are the staff trained in use of relevant systems and process flows? | Yes/No | If no, what is the plan | |
| 2.7 | Are the staff trained to provide after hours advice if required? | Yes/No | If no, what is the plan | |
| **3** | **Contact point** | | | |
| 3.1 | Do you have a plan to monitor the central database for incoming new cases to be contacted? | Yes/No | If no, what is the plan | |
| 3.2 | Have you identified a 24/7 single point of contact (email, phone, contact position for escalation for QAS, Queensland Health Contact Centre, HHS (Hub and Spoke), Public Health Units, Department of Health, GPs, community members and other services involved in the care of people with COVID-19? The phone number must have a message service or forward to another number if busy | Yes/No | If no, what is the plan | |
| 3.3 | Have you developed an escalation guideline for instances where unable to contact nominated person/area? | Yes/No | If no what is the plan | |
| **4** | **Consumer engagement and communication** | | | |
| 4.1 | Have you provided information to consumers within your HHS regarding the model and actions expected of them should they become or be caring for a COVID-19 positive person? | Yes/No | If no what is the plan | |
| 4.2 | Are you able to provide interpreter services where required and use the National Relay Service for hearing or speech impaired people? | Yes/No | If no, what is the plan | |
| 4.3 | Do you have dedicated support persons for First Nations people? | Yes/No | If no, what is the plan | |
| 4.4 | Are you able to provide culturally safe care, communication, and management of First Nations people, ensuring and prioritising the deployment of experienced First Nation Nurses, Health Worker, and Indigenous Liaison Officer roles | Yes/No | If no, what is the plan | |
| **5** | **Public Health Unit liaison** | | | |
| 5.1 | Have you communicated with your local Public Health Unit regarding their role in the model within your HHS, and identified actions required for any escalation of care for a person? | Yes/No | If no, what is the plan | |
| 5.2 | Have you developed local public health processes on how to manage risks and public health activities at the local level? | Yes/No | If no, what is the plan | |
| **6** | **Record keeping / Discharge summaries** | | | |
| 6.1 | Do you have a process in place for recording clinical details and assigning clinical risk? | Yes/No | If no, what is your mitigation strategy | |
| 6.2 | Do you have a process for completion of a discharge summary and uploading to The Viewer within 24 hours of discharge? | Yes/No | If no, what is your mitigation strategy | |
| **7** | **Risk stratification and management** | | | |
| 7.1 | Do you have a Monoclonal antibody therapy (MAB) pathway / management process for the HHS and how this will interact with virtual care? | Yes/No | If no, what is your mitigation strategy | |
| 7.2 | Do you have a process to de-escalate people to the COVID well stream, where care needs have reduced? i.e. back via Healthdirect Australia? | Yes/No | If no, what is your mitigation strategy | |
| **8** | **At risk populations**  Where you have determined within your HHS that some people / cohorts are not suitable to be managed via Healthdirect Australia, e.g. First Nations, CALD, rural and remote, people with a disability, elderly, medical risk, social risk, people requesting/requiring additional support, unvaccinated, infants, immunocompromised, cognitively impaired): | | | |
| 8.1 | Have you identified people that require an individualised / HHS approach due to their geographical location e.g. persons residing in remote communities, prisons, or other factors? | Yes/No | If yes, if possible provide post codes or addresses | |
| 8.2 | Have you developed a process for local management of at-risk populations? | Yes/No | If no, what is your mitigation strategy | |
| 8.3 | Have you developed a process for outbreak management in these communities / cohorts? This includes when to engage/disengage the Healthdirect Australia model of care during a declared outbreak | Yes/No | If no, what is your mitigation strategy | |
| 8.4 | Where people are excluded from the Healthdirect Australia model of care, have you developed a process of how to undertake the following:   - Monitor incoming notifications - Conduct the initial survey (patient demographic, medical, social, public health) - Complete / attempt first location activities of people where contact details are incorrect and provide updated information in the central data platform - Script to seek the persons consent to collect data, consent to share data and consent to treatment in the community - Process to record details of people that do not consent to share data and consent to treatment in the community - Process on how to advise of any requirements they must meet including provision of information for public health purposes under the *Public Health Act 2005*, including requirement to isolate - Process of how to complete initial assessment to identify care needs and public health risk factors - Process for how to return all survey data (demographic, public health and clinical) to the central data platform - Guideline for when to call 000 for people assessed as having urgent care needs - Refer the person to the appropriate care and management stream (COVID well (refer back to Healthdirect Australia, or allocation to COVID of concern or Hospital care streams) - Escalation processes to nominated HHS delegate for hot handover for HHS community care, details of people assessed as requiring treatment within hospitals developed - Process for how to identify and provide any interpreter requirements, including use of the National Relay Service for hearing or speech impaired people - Identify interpreter capacity | Yes/No | If no, what is your mitigation strategy | |
| **9** | **Spoke/Hub Relationship** | | | |
| 9.1 | Have you developed a relationship between your HHS / facility and another HHS / facility in a Spoke / Hub type relationship that details the following?   - Hub / Spoke support relationship identified and agreed - Support requirements from the Hub identified and communicated - Triggers for commencement of Spoke / Hub process - Clear contact / communication requirements between Hub and Spoke - Clear process for clinical information exchange as required | Yes/No |  | |

Appendix 2

**Table 4: Triage Questions**

Assess patient current condition and underlying health conditions and co-morbidities to assess suitability for care under virtual model.

1. Confirm patient’s name, age, address
2. What symptoms do they have?

(Legend: Red = High Risk, Orange= Moderate Risk, Black = Other)

| **Symptom** | **Tick** | **Action** |
| --- | --- | --- |
| New breathlessness (see appendix for severity guide) |  | Requires admission |
| Fatigue (see appendix for severity guide) |  | Requires admission |
| Confusion/altered level of consciousness |  | Requires admission |
| Chest pain |  | Requires admission |
| Cold/Clammy |  | Requires admission |
| Collapse/dizziness on standing up |  | Requires admission |
| Diarrhoea >4x/day |  | Requires admission |
| Fever > 38C |  | Consider in person review |
| Productive cough |  | Consider in person review |
| Significant Pain/Myalgia (see appendix for severity guide) |  | Consider in person review |
| Mild cough or URTI symptoms |  |  |
| Mild GI symptoms |  |  |

1. Are there high risk social factors?

| What is your home situation like?  Rule out share houses or boarding house, shared amenities in same household of uninfected persons |  |
| --- | --- |
| What is your access to a phone?  Do they have a phone and phone credit, alternative phone number if possible |  |
| Are they at risk for violence, abuse or neglect in their household during isolation? |  |
| Do they suffer from any major mental illness?  Ask if they see a psychiatrist and what for/diagnosis. Consider referral to Mental Health for telehealth support during period of isolation |  |
| Do they require an interpreter to communicate? |  |
| Do they require support for personal ADLS and who provides this support? Family vs service provider |  |
| Will they be able to access food, fluids, supplies during their period of isolation including medications? | Inform patient of Community recovery hotline 1800 173 349  Consider Social Worker referral |
| Do they consent to remain in isolation based on public health orders?  Are they aware that they need to remain in isolation until they receive public health clearance from their Virtual COVID team? |  |

1. Do they have medical risk factors?

(Legend: Red = High Risk, Orange= Moderate Risk, Black = Other)

| **Medical Risk Factor** | **Tick** | **Action** |
| --- | --- | --- |
| Pregnancy |  | Consult with Obstetrics team regarding admission vs Virtual monitoring |
| Organ transplant on immune suppressive therapy |  | Requires admission |
| Immune suppressive therapy for graft vs host disease |  | Requires admission |
| Haematopoeitic stem cell transplant in last 2 years |  | Requires admission |
| HIV infection with CD4 T lymphocyte count <200 cells/mm^3^ |  | Requires admission |
| Undergoing chemotherapy |  | Requires admission |
| Age >65 (>55 ATSI) |  |  |
| Chronic lung disease |  |  |
| Cardiovascular disease (excluding hypertension) |  |  |
| Chronic kidney disease |  |  |
| Diabetes Mellitus |  |  |
| Liver disease |  |  |
| Significant frailty or disability |  |  |
| Active cancer |  |  |
| Other immunosuppressive therapy |  |  |
| Other comorbidities (please list) |  |  |

1. Have they had 2 vaccine doses (2^nd^ dose > 2 weeks ago)?

| 1 dose only |  |
| --- | --- |
| 2 doses but < 2 weeks ago |  |
| 2 doses >2 weeks ago or 3^rd^ dose |  |

Appendix 3

**Table 5: Checklist to commence patient admission to the virtual ward**

| **Actions** | **Tick** |
| --- | --- |
| Patient is risk stratified to risk categories (double check with CN/doctor) to determine frequency of interventions on MyCareManager |  |
| Identify patients requiring face-to-face review:   - Unvaccinated + High Risk Medical Factors or >2 Moderate Risk Medical Factors or significant disease in any Moderate Risk Medical Factors - Patients with Moderate Risk Symptoms (High risk symptoms should be referred for admission to Designated COVID Hospital) |  |
| If patient is suitable for Sotrovimab infusion (see pathway and appendix). Notify Medical officer as may be referred to Designated COVID Hospital for Sotrovimab infusion |  |
| Frequency of patients surveys and observations is selected on MyCareManager for the duration of their isolation |  |
| Check with patient when equipment has been delivered. Alert Telstra Health if not delivered during predicted time frame |  |
| Patient information pack has been provided, email vs hard copy delivery |  |
| Preferred contact method is noted – preferably with alternative phone number |  |
| Consideration for consults: Obstetrics, Mental Health, Social Worker, Indigenous Liaison support |  |

Appendix 4

**Table 6: Guide to classify dyspneoa characteristics & severity**

This questionnaire is only to quantify severity. All patients reporting **objective or subjective symptoms of dyspnoea** are to be referred to hospital for admission and escalation of management/monitoring.

Questions should be asked only if dyspnoea is new or worse than baseline.

Questions are based on how they have felt in the last 12 hours.

| **Dyspnoea Characteristics & Severity** | **Answer** |
| --- | --- |
| How short of breath have you been? | Very mild  Mild  Moderate  Severe  Very severe |
| Intensity of shortness of breath | When I had shortness of breath, it felt:   - Very mild - Mild - Moderate - Severe - Very severe |
| Frequency of shortness of breath | I felt short of breath:   - Once in the last 12 hours - Several times in the last 12 hours - Persistently over the last 12 hours |
| Duration of shortness of breath | When I had shortness of breath, it lasted:   - Only for a moment - For a few minutes (<10 minutes) - For a long time (>30 minutes) - Continuously |

Appendix 5

**Table 7: Guide to classify fatigue and pain**

**Fatigue**

Questions should be asked only if fatigue is new or worse than baseline.

Questions are based on how they have felt in the last 12 hours.

| Dressing without help | Fatigue:   - Very mild - Mild - Moderate - Severe - Very severe |
| --- | --- |
| Walking 50 steps/paces on flat ground at a normal speed without stopping | Fatigue:   - Very mild - Mild - Moderate - Severe - Very severe |
| Walking up 10 stairs (1 flight) without stopping | Fatigue:   - Very mild - Mild - Moderate - Severe - Very severe |
| Carrying something weighing less than 5 lbs (about 2 kg, like a laundry basket) from one room to another | Fatigue:   - Very mild - Mild - Moderate - Severe - Very severe |

**Pain Survey**

Questions should be asked only if pain is new or worse than baseline.

| **Pain Intensity Survey** | **Answers** |
| --- | --- |
| Where is the location of your pain? |  |
| How long have you had this pain? | _____ hours  _____days  A long time but getting worse |
| How would you rate your pain on average? | Mild pain Moderate pain  Severe pain Very severe pain  Worst imaginable |
| What is your level of pain right now? | No pain  Mild  Moderate Severe Very severe |
| Is your pain medication(s) helping to control your pain? | Always  Often  Sometimes Rarely  Never |

Appendix 6

**Criteria for Sotrovimab in patients with COVID-19**

Sotrovimab may be considered for the following adult patients with a current diagnosis of COVID-19:

- - with symptom onset of no more than 5 days AND
  - who do not require oxygen AND
  - who have reduced immunity to COVID-19 e.g. not vaccinated, not fully vaccinated# or immunosuppressed* (irrespective of vaccine status) AND
  - who have one or more of the following risk factors:
  - Diabetes (requiring medication)
  - Obesity (BMI >30 kg/m2)
  - Chronic kidney disease (i.e., **eGFR <60 mL/min/1.73m2)
  - Congestive heart failure (New York Heart Association (NYHA) class II or greater)
  - Chronic obstructive pulmonary disease (history of chronic bronchitis, chronic obstructive lung disease, or emphysema with dyspnoea on physical exertion)
  - Moderate-to-severe asthma (requiring an inhaled steroid to control symptoms or prescribed a course of oral steroids in the previous 12 months)
  - Age ≥55 years (or age >35 years if Aboriginal and/or Torres Strait Islander)

Not fully vaccinated includes adults who have had only 1 vaccine dose or those whose 2nd dose was administered less than 2 weeks prior.

For the purposes of this guidance, immunosuppressed patients are those that:

a) have a primary or acquired immunodeficiency such as haematological neoplasms, are post-transplant [solid organ (on immunosuppressive therapy) or haematopoietic stem cell transplant within 24 months], or have HIV/AIDS or other significant immunocompromising condition or

b) are on/have been on recent immunosuppressive therapy such as chemotherapy, radiotherapy, high dose corticosteroids (equivalent to 20 mg or more of prednisone per day for 14 days or more) or are on biologic immunotherapies including most disease-modifying anti-rheumatic drugs (DMARDs).
